# Targeting spinal cord perfusion pressure in acute spinal cord injury through cerebrospinal fluid drainage: A prospective multi-center clinical trial

**DOI:** 10.1371/journal.pmed.1004925

**Published:** 2026-02-05

**Authors:** Cameron M. Gee, Angela Tsang, Miko McKenzie, Lise Belanger, Leanna Ritchie, Tamir Ailon, Charlotte Dandurand, Scott Paquette, Raphaële Charest-Morin, Nicolas Dea, John Street, Charles G. Fisher, Jefferson Wilson, Anthony DiGiorgio, Jean-Marc Mac-Thiong, Sean Christie, Jamie Wilson, Christian Ricks, David Okonkwo, Brian K. Kwon

**Affiliations:** 1 International Collaboration on Repair Discoveries (ICORD), Faculty of Medicine, University of British Columbia, Vancouver, British Columbia, Canada; 2 Department of Orthopaedics, Faculty of Medicine, University of British Columbia, Vancouver, British Columbia, Canada; 3 Vancouver Spine Research Program, Vancouver General Hospital, Vancouver, British Columbia, Canada; 4 Division of Neurosurgery, Department of Surgery, Faculty of Medicine, University of British Columbia, Vancouver, British Columbia, Canada; 5 Department of Neurosurgery, University of Toronto, Toronto, Ontario, Canada; 6 Department of Neurological Surgery, University of California San Francisco (UCSF), San Francisco, California, United States of America; 7 Faculty of Medicine, University of Montreal, Montreal, Quebec, Canada; 8 Division of Neurosurgery, Dalhousie University, Halifax, Nova Scotia, Canada; 9 Department of Neurosurgery, University of Nebraska, Lincoln, Nebraska, United States of America; 10 Medical University of South Carolina, Charleston, South Carolina, United States of America; 11 Department of Neurosurgery, University of New Mexico, Albuquerque, New Mexico, United States of America; 12 Department of Neurological Surgery, University of Pittsburgh, Pittsburgh, Pennsylvania, United States of America; Barts and the London School of Medicine & Dentistry Queen Mary University of London, UNITED KINGDOM OF GREAT BRITAIN AND NORTHERN IRELAND

## Abstract

**Background:**

The hemodynamic management of acute spinal cord injury (SCI) aims to improve perfusion and mitigate ischemic secondary injury to the injured spinal cord, traditionally through the augmentation of mean arterial pressure (MAP). Recently, there has been interest in managing spinal cord perfusion pressure (SCPP)—the difference between MAP and intrathecal pressure (ITP) —after acute SCI. SCPP may be more physiologically relevant than MAP for neurologic recovery after traumatic SCI. Drainage of cerebrospinal fluid (CSF) through a lumbar intrathecal catheter to reduce ITP and increase SCPP is commonly performed to reduce the risk of ischemic paralysis in thoracoabdominal aortic aneurysm (TAAA) surgery. We investigated a protocol for CSF drainage through intrathecal catheters to maintain SCPP ≥65 mmHg in participants with acute traumatic SCI. We sought to determine if managing SCPP was associated with better neurologic recovery compared to traditional MAP targets.

**Methods and findings:**

Fifty-eight participants with acute SCI (51 ± 19 years, 46M/12F) were enrolled across eight North American sites between August 2019 and May 2024 into this prospective single-arm multi-center clinical trial of CSF drainage for SCPP management (NCT03911492). Data were compared to data from a historical cohort of 86 participants (44 ± 19 years, 72M/14F) who had intrathecal catheters inserted for SCPP measurement only; these participants were managed according to conventional MAP guidelines with a target MAP of 85–90 mmHg (NCT01279811). MAP, ITP, SCPP, intrathecal waveform morphology, vasopressor use, and CSF drainage volume were reported for up to 7 days following SCI. Fifteen participants in the intervention group were lost to follow-up. Neurological assessments at enrollment and 6-months post-SCI were compared. The investigator team ended the trial when it was clear that adherence to the protocol was inconsistent across study sites. Participants managed according to the SCPP management protocol had an intrathecal catheter in place 138 hours (95% CI [129,147]) and 495cc (95% CI [350,641]) of CSF drained. No CSF was drained from seven participants. There were no significant differences in hemodynamic measures such as ITP and SCPP between groups, indicating that the SCPP management protocol did not alter the hemodynamic management. Subsequently, there were no differences in measures of neurological recovery between participants managed according to SCPP management protocol and conventional MAP guidelines (*p* = 0.897). Participants managed according to an SCPP target had more ITP waveform recordings noted as dampened or fully pulsatile suggesting a patent subarachnoid space *(p* = 0.006) and were administered vasopressors on fewer hourly observations *(p* = 0.004). Six reported adverse events were probably related to the intervention. Adherence to a protocol for managing SCPP through CSF drainage across multiple sites was challenging.

**Conclusions:**

Ultimately, our protocol resulted in little CSF being drained, limited modification of ITP and SCPP, and no effect on neurological recovery. The relationship between CSF drainage volume and change in ITP was surprisingly unclear. This study revealed that draining CSF is more complex in traumatic SCI than in TAAA surgery patients. Future efforts to reduce ITP through CSF drainage likely need to address the occlusion of the subarachnoid space at the injury site through aggressive surgical decompression techniques.

## Author summary

**Why was the study done?:**

- The hemodynamic management of acute spinal cord injury is one of the mainstays of early management, with mean arterial pressure guidelines existing for almost 20 years. However, whether mean arterial pressure guidelines are the best target for enhancing the likelihood of recovery has been questioned.
- Recent evidence indicates that spinal cord perfusion pressure—the difference between mean arterial and intrathecal pressure—may be a better predictor of neurological recovery than mean arterial pressure following spinal cord injury.
- We aimed to test whether actively managing spinal cord perfusion pressure through a combination of mean arterial pressure augmentation and cerebrospinal fluid drainage was associated with better neurological recovery compared to conventional hemodynamic management.

**What did the researchers do and find?:**

- We recruited 58 participants with acute spinal cord injury across 8 North American sites and monitored them for one-week post-injury. Participants had a lumbar intrathecal catheter inserted during surgery to monitor spinal cord perfusion pressure and to drain cerebrospinal fluid to reduce intrathecal pressure.
- Data were compared to a historical cohort of 86 participants who were managed according to conventional mean arterial pressure guidelines for five days post-injury.
- Ultimately, cerebrospinal fluid drainage was not associated with differences in spinal cord perfusion pressure between groups and there was no difference in neurological recovery between groups.

**What do these findings mean?:**

- The relationship between the volume of cerebrospinal fluid drained and the change in intrathecal pressure was surprisingly unclear.
- These findings highlight the importance of surgical decompression in drainage studies and suggest that alternative drainage protocols may be needed.
- This study highlights the challenges in implementing multi-center studies in the critical care setting and with adhering to protocols across multiple sites.

## Abstract 3

Cameron M. Gee and colleagues not only report whether targeting spinal cord perfusion pressure via cerebrospinal fluid drainage after acute traumatic spinal cord injury leads to better neurologic recovery than standard mean arterial pressure targets, but also highlight the challenges of conducting multicenter studies in critical care settings and of adhering to protocols across multiple sites.

## Introduction

A traumatic spinal cord injury (SCI) is a catastrophic event for which there continues to be few treatment options available to improve neurologic recovery. Aggressive hemodynamic management has been widely adopted as one of the few options available in the early treatment of acute SCI, with the goal of improving perfusion of the injured cord to mitigate ischemic secondary injury [1]. Clinical practice guidelines such as those of the American Academy of Neurological Surgeons and Congress of Neurological Surgeons from 2013 [2] and the more recently published guidelines from AO Spine and the Praxis Spinal Cord Institute in 2024 [3] recommend the augmentation/maintenance of mean arterial blood pressure (MAP) to specific levels during the first week post-injury.

The systematic review on hemodynamic management that informed the AO Spine/Praxis 2024 guidelines concluded that the overall quality of the relevant literature on MAP augmentation was very weak, resulting in an uncertain relationship between specific MAP targets and neurologic recovery [4]. A potential reason for this uncertainty is that MAP alone may not be the best measure of how well the injured spinal cord is perfused. Rather, spinal tissue perfusion may be better reflected by spinal cord perfusion pressure (SCPP), calculated as the difference between MAP and the pressure of the cerebrospinal fluid (CSF) around the cord, which we refer to as “intrathecal pressure” (ITP). This concept is analogous to the hemodynamic management of acute traumatic brain injury where the focus is primarily on maintaining appropriate cerebral perfusion pressure—equal to the difference between MAP and intracranial pressure [5].

To this end, our group performed a prospective *non-interventional* trial entitled “CAMPER” (the Canadian Multicentre CSF Pressure and Biomarker Study; *ClinicalTrials.gov* ID: NCT01279811) in which lumbar intrathecal catheters were utilized to monitor ITP—and calculate SCPP—in participants with acute SCI. The CAMPER trial found that SCPP held a stronger association with neurologic recovery than did MAP [6]. Further, maintaining SCPP at ~65 mmHg was associated with improved neurologic outcome following traumatic SCI [7].

While MAP augmentation to increase SCPP has been the mainstay of conventional hemodynamic management for acute SCI, SCPP may also be increased by reducing ITP through CSF drainage [8]. The concept of draining CSF to reduce ITP and increase SCPP is a widely accepted neuroprotective intervention in the setting of open thoracoabdominal aortic aneurysm (TAAA) repair surgery wherein the spinal cord’s blood supply is inherently vulnerable [9–11]. Applying such an approach in acute SCI may allow for reduced use of vasopressors, which have been linked to increased cardiovascular complications [12] and hemorrhage at the injury site [13].

The current study entitled “CASPER” (The Canadian-American Spinal Cord Perfusion and Biomarker Study) was initiated following insights about SCPP monitoring in acute SCI from the CAMPER trial, and was a prospective single-arm *interventional* trial to examine the active management of SCPP through ITP reduction via CSF drainage. The key objectives of the CASPER trial were to determine: (a) whether actively maintaining SCPP ≥65 mmHg with a combination of MAP augmentation and CSF drainage promotes better neurologic recovery than routine hemodynamic management that focuses solely on MAP augmentation; (b) if actively maintaining SCPP ≥65 mmHg with a combination of MAP augmentation and CSF drainage will allow for a reduction in the usage of vasopressors in acute SCI; (c) the feasibility of draining CSF to reduce ITP in the acute post-injury setting, when the cord may be swollen against the dura causing subarachnoid space (SAS) occlusion at the injury site; and (d) if there are complications associated with the installation of the intrathecal catheter and drainage of CSF in individuals with acute SCI.

## Methods

### Study design and participants

**Fig 1:**
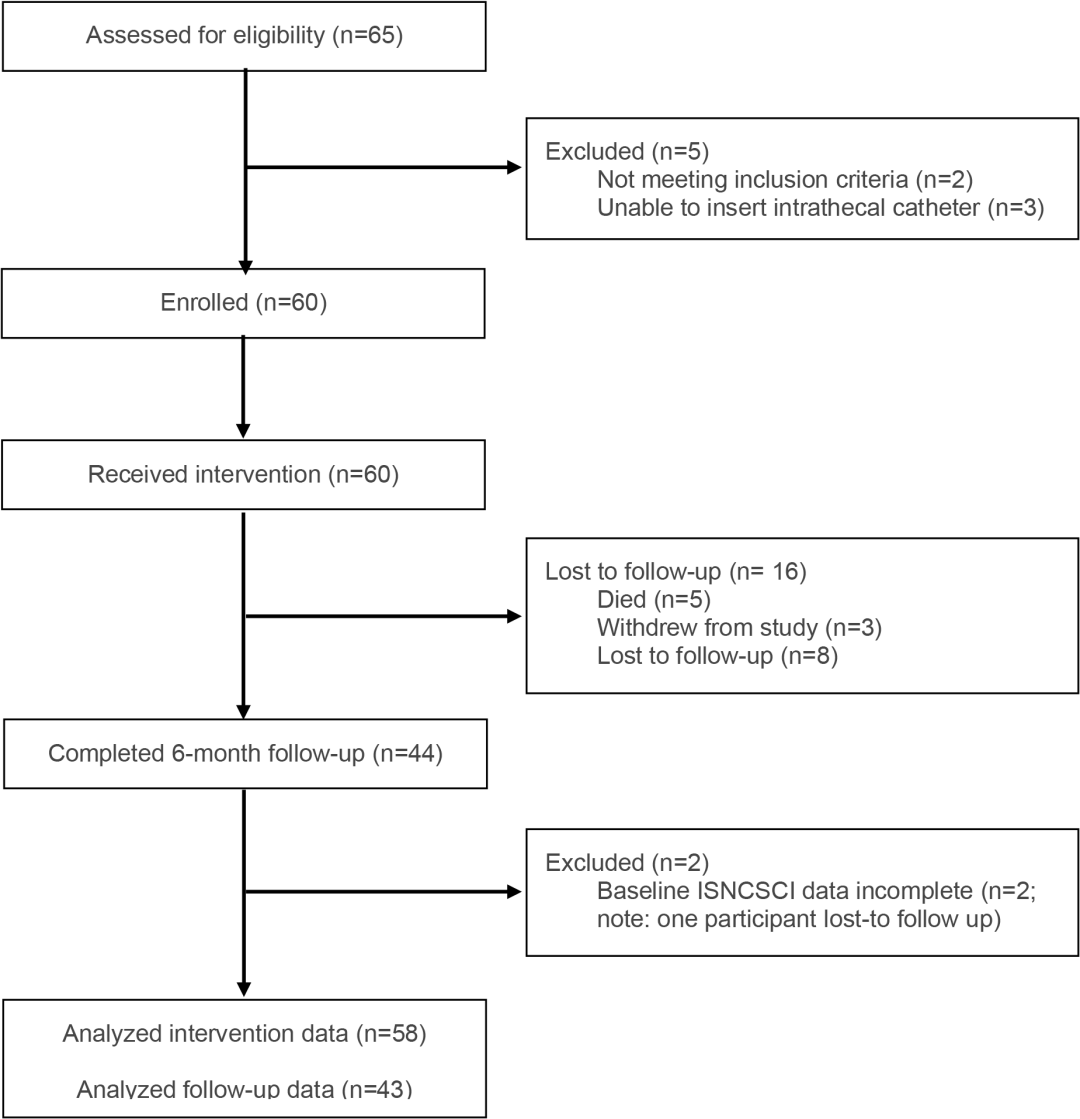
CONSORT flow diagram. Summary of participants assessed for eligibility, enrolled, receiving intervention, completing 6-month follow-up, and analyzed as part of CASPER cohort. *Abbreviations:* ISNCSCI, International Standards for the Neurological Classification of Spinal Cord Injury.

The CASPER trial (*ClinicalTrials.gov* ID: NCT03911492) was a prospective multi-center single-arm clinical trial conducted at four Canadian and four American sites (S1 Table). Multi-center recruitment for the CASPER trial occurred between August 2019 and May 2024. A CONSORT flow diagram is provided in Fig 1.

Participants were enrolled in CASPER with the following inclusion criteria: (1) non-penetrating SCI with surgical or non-surgical treatment; (2) American Spinal Injury Association Impairment Scale (AIS) grade A (motor- and sensory-complete), B (motor-complete and sensory-incomplete), or C (motor-incomplete) SCI upon presentation; (3) spinal injury between C1 and T12; (4) a lumbar intrathecal catheter could be inserted within 48 hours of injury; (5) an initial blood sample could be collected with 24 hours of the injury; (6) ≥17 years of age. Participants were excluded from enrollment if they (1) had associated trauma that would interfere with outcome assessments; (2) had isolated radiculopathy or cauda equina injury; (3) were pregnant; (4) pre-existing neurodegenerative disorder/s, autoimmune disorder/s, or thromboembolic disease; or (5) any other medical condition that might interfere with participant safety.

### Procedures

#### Hemodynamic management protocol for the CASPER trial.

The basic interventional approach in the CASPER trial was to utilize an indwelling lumbar intrathecal catheter to measure ITP and drain CSF to a target pressure of 15 mmHg, rather than solely relying on MAP augmentation to maintain SCPP ≥65 mmHg for up to seven days post-injury. A flowchart informed critical care bedside nurses on the hemodynamic management of participants enrolled in the CASPER trial (S1 Fig). The clinical trial protocol was approved by the Clinical Research Ethics Board affiliated with each study site and all participants provided written informed consent if they were able to and oral informed consent if not (S1 Table).

While the focus of the trial was on SCPP, MAP augmentation was implemented if the participant was hypotensive (i.e., systolic blood pressure <90 mmHg). When necessary, elevation in systolic blood pressure was achieved by volume replacement followed by vasopressors as needed.

If the participant was normotensive but their SCPP <65 mmHg, critical care bedside nurses were to examine ITP and the morphology of the ITP waveform. Based on previous data [14] we considered a flat ITP waveform to reflect an occluded SAS around the injured spinal cord (so long as the catheter itself was not kinked/occluded), and a pulsatile or ‘dampened pulsatile’ waveform reflected an SAS sufficiently patent at the injury site so as to transmit the pulsation of pressure waves from the brain to the lumbar cistern [15]. A patent SAS at the injury site was considered to be important in establishing a valid measurement of ITP and for being able to continuously drain CSF. As a result, if SCPP was <65 mmHg and ITP >15 mmHg with a dampened or pulsatile waveform, the lumbar drain was opened, allowing for CSF drainage via gravity through a pressure-mediated system, in order to achieve an SCPP ≥65 mmHg.

If SCPP <65 mmHg but ITP ≤15 mmHg, the lumbar drain would remain closed and MAP would be augmented to achieve the target SCPP goal. If the ITP waveform was determined to be flat (suggesting a non-patent SAS and an inaccurate ITP measurement), regardless of the ITP value, a target MAP of ≥85 mmHg would be achieved rather than using an SCPP goal. For MAP augmentation, the CASPER protocol did not impose the use of a specific vasopressor, and this choice was left to the individual institution/clinician.

#### Lumbar intrathecal catheter insertion and CSF drainage.

The lumbar intrathecal catheter was inserted pre-operatively according to standard practice. The catheter was advanced 5–15 cm into the SAS and connected to a drainage system leveled at the mid-axillary line. If ITP was >15 mmHg and the ITP waveform was pulsatile to some extent, the drain was opened with the pressure gradient set to 15 mmHg to allow for drainage of CSF via gravity. The drain was then to be closed each hour to re-check the ITP measurement and the CSF drainage volume was documented. If ITP remained >15 mmHg with a pulsatile waveform, the drain was re-opened. This pattern of closing the drain, checking the ITP, and then re-opening the drain for CSF drainage was necessary as the drainage system did not allow simultaneous measurement of ITP while draining CSF (i.e., the drain must be closed in order to obtain a pressure measurement), and to prompt bedside nurses to assess the participant’s neurologic and clinical status. Each site adopted one of the following approaches to CSF drainage:

(1) volume limited: drain to a maximum volume of 30 cc of CSF within the hour, without setting a specific target pressure.
(2) pressure-limited: set the drain to 15 mmHg and open it, with no restriction on the amount of CSF drainage per hour.
(3) hybrid volume-pressure limited: set the drain to 15 mmHg and open it, but limit the amount of CSF drained within one hour to 30 cc.

This pragmatic approach permitted centers to employ CSF drainage techniques of familiarity and with the greatest track record of safety and effectiveness.

### Outcomes

#### Demographics.

Upon enrollment, baseline demographic data were collected on participant’s age, sex, previous medical history, medication history, mechanism of injury, and injury diagnosis.

#### Hemodynamic monitoring.

MAP, ITP, and SCPP were recorded by bedside nurses either in the intensive care unit or within a high-acuity stepdown unit (in accordance with institutional practices). Hemodynamic indices were monitored in real time and displayed on bedside monitors. MAP was measured by an arterial line placed in the radial artery and ITP by the above-mentioned intrathecal catheter. SCPP was calculated as the difference between MAP and ITP. MAP variability and the distribution of MAP recordings is reported as <85, 85–90, and >90 mmHg based on recommendations for hemodynamic management at the time the trial was initiated [2,16]. SCPP distribution is reported as the percentage of recordings where SCPP was ≥65 mmHg. To assess the effect of the “overall exposure” to SCPPs that fell below the 65 mmHg threshold, we calculated an “area under the curve” as the sum of all measures <65 mmHg divided by the total number of SCPP measures (because not all participants were monitored for an equal length of time or had the same number of SCPP measures).

#### Vasopressor administration.

Administration and dose of vasopressors, including norepinephrine, epinephrine, phenylephrine, dopamine, and vasopressin was to be reported each hour. The choice of vasopressor was left to clinician preference, and dosages were determined based on what was felt to be clinically indicated for each participant. Rather than normalize all the different vasopressors and their dosages into a norepinephrine equivalent, we calculated “vasopressor usage” as the percentage of the total observations during which participants received one or more vasopressor.

#### Associations between cerebrospinal fluid drainage and changes in intrathecal pressure.

The volume of CSF drained was to be recorded hourly by bedside nurses. At the UBC/Vancouver General Hospital (VGH) study site, we also had the ability to collect more granular hemodynamic data than other sites using a multimodal neuro-intensive care monitoring system (ICM+; Cambridge Enterprise, Cambridge, UK). This system collected hemodynamic data at 100 Hz and reported data averaged each minute. Given this monitoring ability, in a subset of six consecutive participants enrolled at the UBC/VGH study site, bedside nurses documented the exact time the lumbar drain was opened and closed, thus allowing us to examine the temporal relationship between CSF drainage and ITP. Here, it is important to note that one cannot measure ITP while the drain is open and draining CSF. Hence, to determine how CSF drainage alters ITP, the drain must be closed, the ITP recorded, and then the drain opened to allow for CSF drainage. After this, the drain must be closed again to check the ITP. Pre-drainage ITP was determined as the minute prior to the start of CSF drainage and post-drainage ITP as the average of up to 10 min following the end of drainage. This change in ITP between pre-drainage and post-drainage measurements could then be compared with the amount of CSF that was drained during the time between ITP measurements. Only “drainage events” when the drain was open between 50 and 70 min were included in this analysis in order to interpret how drainage over the course of approximately one hour impacted ITP. This data was collected over the entire monitoring period of all six participants.

#### Intrathecal pressure waveform morphology.

**Fig 2:**
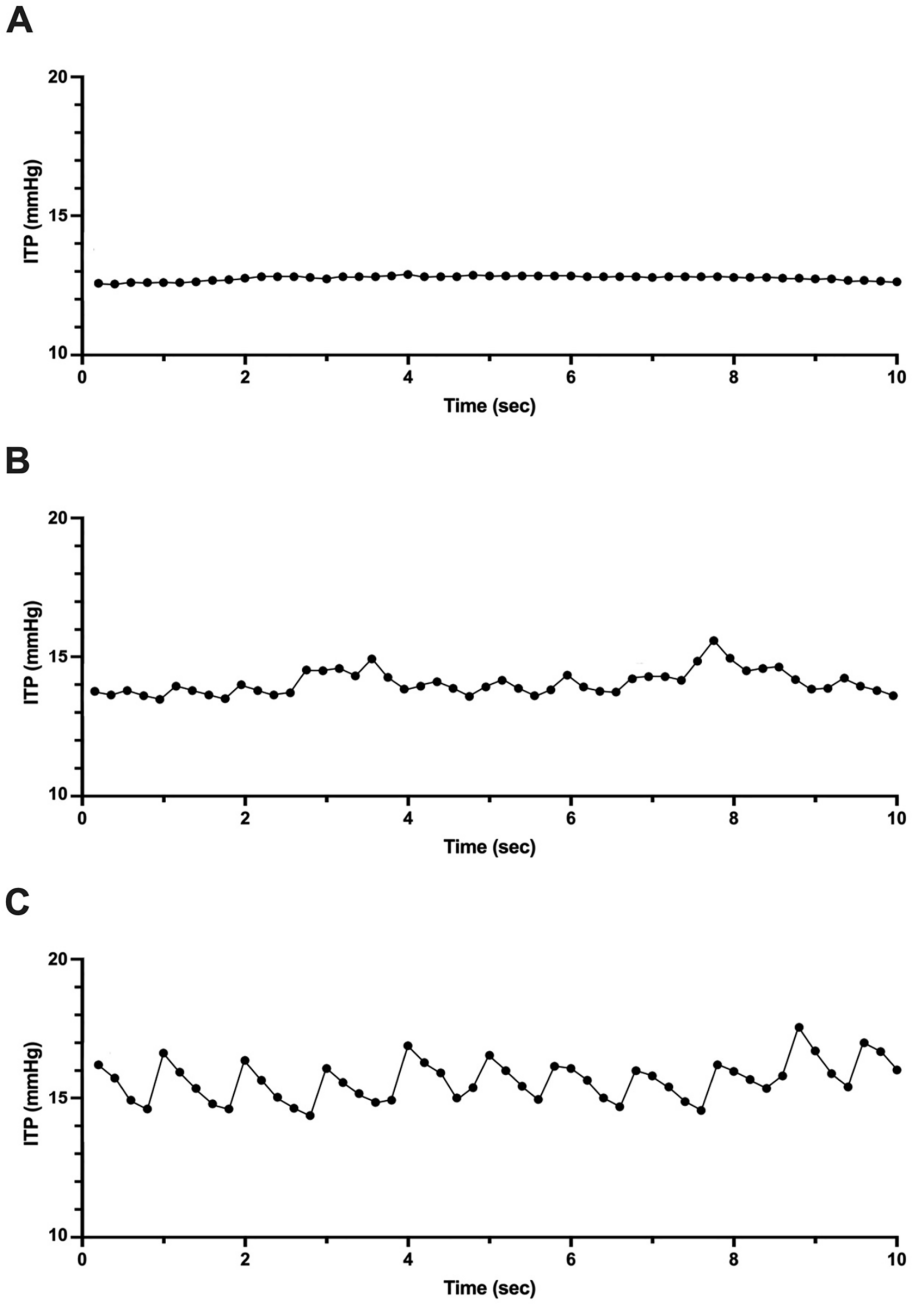
Representative intrathecal pressure waveforms. Waveforms recorded by indwelling lumbar intrathecal catheter may be categorized as **(A)** flat, **(B)** dampened pulsatile, or **(C)** pulsatile. *Abbreviations:* ITP, intrathecal pressure.

Critical care and high acuity trained bedside nurses were trained on how to interpret the ITP waveform morphology as being either (a) flat, (b) dampened pulsatile, or (c) fully pulsatile (Fig 2) and documented the morphology. As stated earlier, the ITP waveform was considered as a form of “biomarker” for the patency of the SAS, with a flat waveform considered to be indicative of an occluded SAS whereas dampened pulsatile and fully pulsatile waveforms reflected degrees of SAS patency [14].

#### Neurological assessment.

The International Standards for the Neurological Classification of Spinal Cord Injury (ISNCSCI) [17] was used to determine the level and completeness (i.e., AIS grade) of injury as well as motor scores through the key muscle groups. Baseline neurological assessments were performed at enrollment into the study, and the ISNCSCI examination was formally repeated at 6-months post-injury for the determination of neurologic recovery. Recovery was operationalized as positive AIS grade conversion and/or an improvement in motor score equal to seven points or greater. A seven-point motor score change was chosen as previous studies suggest this to be a meaningful change in motor function and one that is realistically achievable with early surgical decompression in acute SCI [18,19].

### Blood samples

Samples were taken three times each day for 7 days following SCI from each participant enrolled in CASPER for biomarker analysis. These data will be reported elsewhere.

### Data storage

All participant data collected at each study site was coded with a unique study ID. All documents linking the participant’s personal identifiers to the study ID number were accessible only by the local study site research team. De-identified data was entered into the Praxis Spinal Cord Institute’s Global Research Platform; a web-based, secure data collection, storage, and research management system.

### The CAMPER trial hemodynamic management protocol (“Historical Control”)

We used data collected during the CAMPER trial (ClinicalTrials.gov NCT01279811) as a ‘historical control’ to the CASPER participants. In the CAMPER trial, lumbar intrathecal catheters were inserted but used only for monitoring ITP and SCPP, not for actively draining CSF. The protocol and hemodynamic data for the CAMPER prospective *non-interventional* trial has been described previously [6]. Briefly, lumbar intrathecal catheters were inserted in acute SCI participants for five days to measure ITP and SCPP, and the participants had standard hemodynamic management with volume replacement and vasopressor to maintain MAP at 85–90 mmHg, in accordance with the previously established guidelines from 2013 [2]. The key research question was how ITP, SCPP, and MAP related to neurologic outcome. The CAMPER trial was conducted from January 2012 to October 2019 and was conducted at some of the same sites that subsequently participated in CASPER (Vancouver, Montreal, Toronto, Halifax, and San Francisco) as well as University of Western Ontario (London). Because the observations about the relevance of SCPP in CAMPER led directly to the rationale and design of CASPER, we describe the results of CAMPER here as a historical control to provide context to the findings of the CASPER trial.

### Sample size calculation

We embarked on the CASPER study with the intent of examining whether active SCPP management with CSF drainage resulted in improved neurologic function as compared to conventional MAP management in the CAMPER study. The primary outcome measure was the change in total motor score at 6-months post-injury (a time point commonly used in acute SCI clinical trials). In doing a sample-size calculation, we estimated a seven-point improvement in motor score determined by the ISNCSCI exam to be clinically meaningful given what had been observed to be achievable with early surgical decompression in acute SCI. A sample size calculation indicated the need to enroll ~74 patients with cervical SCI, based upon a standard deviation of 11 points, reflecting *α* = 0.0499 and *β* = 0.806. Anticipating that we would enroll patients with thoracic SCI who would likely have less motor score change and additionally some follow-up loss, we set out to recruit 100 participants with acute SCI.

### Statistical analyses

Demographic comparisons between the CASPER and CAMPER cohorts were performed using Pearson’s chi-squared tests. As the trials were not of equal timeframe, linear mixed-effects models with fixed effects for day post-SCI, trial parametrized as a two-level-factor (CASPER and CAMPER), and day×trial were performed to assess changes in outcome measures. Independent samples t-tests compared ITP values between flat and combined dampened or pulsatile waveform morphologies in participants enrolled in CASPER only. Regarding analyses of the subset of participants with minute-by-minute data, a dependent samples *t* test assessed difference between pre- and post-drainage ITP. An exploratory post-hoc linear mixed model with change in pressure as the dependent and drainage as the independent variable assessed the association between CSF drainage and change in ITP. A random intercept and slope were included to account for inter-individual variability. The beta-coefficient denotes the change in ITP for every 1mL of CSF drained during drainage events. All analyses were conducted using IBM SPSS Statistics version 29 (IBM Corp., Armonk, NY, USA) and GraphPad Prism, version 9.1.0 (GraphPad Software, LaJolla, CA, USA). Data are presented as mean (95% confidence interval (CI) [XX,XX]) unless stated otherwise and *p* < 0.05 was considered statistically significant.

## Results

### Participant demographics

Data from 58 participants with acute SCI enrolled in CASPER were compared to 86 historical control participants enrolled in CAMPER. Participant demographics are presented in Table 1. There were no differences in age, sex, injury severity, or motor score at enrollment between groups.

**Table 1:** Participant demographics.

	CASPER *(n = 58)*	CAMPER *(n = 86)*
**Age**	51 ± 19	44 ± 19
**Sex**		
Male	46	72
Female	12	14
**Mechanism of Injury**		
Transport	27	23
Fall	20	33
Sports	8	21
Other	3	9
**Surgical Decompression**		
Anterior	9	22
Posterior	47	58
Unknown	2	6
**Level of Injury**		
Cervical	41	45
Thoracic	17	38
Lumbar	0	3
**AIS Grade**		
A	36	54
B	9	13
C	13	16
D	0	3
**Motor Score**	30 ± 19	33 ± 20

*Abbreviations:* AIS, American Spinal Injury Association Impairment Scale (range: A–E); CASPER, the Canadian-American Spinal Cord Perfusion and Biomarker Study; CAMPER, the Canadian Multicentre CSF Pressure and Biomarker Study.

### Neurological recovery

Six-month follow-up neurological recovery data was collected on 43 and 83 CASPER and CAMPER participants, respectively, and are presented in Table 2. Of the 58 CASPER participants enrolled, four died before the 6-month follow-up time point, three chose to withdraw from the study, and eight were lost to follow-up. There were no significant differences between groups regarding level or severity of injury at 6-month follow-up, or in neurological recovery, AIS grade conversion, motor score, or change in motor score from admission to follow-up (all *p* > 0.274).

**Table 2:** Follow-up neurological recovery.

	CASPER *(n = 43)*	CAMPER *(n = 83)*
**Level of Injury**		
Cervical	27	43
Thoracic	13	35
Lumbar	2	5
N/A	1	
**AIS Grade**		
A	19	34
B	5	15
C	9	8
D	9	26
E	1	0
**AIS Grade Conversion**		
Yes	21	39
No	22	44
**Neurological Recovery**		
Yes	28	55
No	15	28
**Motor Score**	51 ± 27	56 ± 24
**Δ Motor Score**	17 ± 22	22 ± 27

*Abbreviations*: AIS, American Spinal Injury Association Impairment Scale (range: A–E); CASPER, the Canadian-American Spinal Cord Perfusion and Biomarker Study; CAMPER, the Canadian Multicentre CSF Pressure and Biomarker Study. Note: Neurological recovery was operationalized as positive AIS grade conversion and/or an improvement in motor score equal to seven points or greater.

### Cerebrospinal fluid drainage in CASPER participants

The lumbar intrathecal catheter was inserted in CASPER participants for an average of 138 hours (95% CI [129,147]). CSF was not actively drained in the CAMPER study. While the CASPER protocol indicated that the drain should remain in place for seven days, in some participants the drain was removed prematurely for clinical reasons (e.g., the participant was moved from the intensive care unit to a ward where lumbar CSF drains in patients with acute SCI were not routinely managed). As the protocol indicated the lumbar intrathecal catheter be left in place for seven days, the maximum number of hours in which some volume of CSF could theoretically be drained was 168 hours. One participant had the drain left in place for 194 hours (i.e., over 8 days). When examining all of the bedside nurses’ recordings of the volume of CSF drained while the catheter was in place, the volume was “zero” in 68% (5627/8224) of these recordings. In seven participants, no CSF was drained at any point in time that the catheter was in place (i.e., all recordings of the volume of CSF drained were “zero”). So, while the intention was to drain CSF to reduce ITP and increase SCPP, it turned out that CSF was actually drained quite infrequently. In some instances, we noted that no CSF was drained even when the protocol indicated that drainage should occur, with an ITP >15 mmHg and a pulsatile ITP waveform (for example, see S2 Fig). We did note that in some cases, the bedside nurse made multiple recordings of CSF volume drainage during the hour, and so some of the “zero” recordings may be within the same hour that some CSF was later drained. Nonetheless, the overall picture that emerged from the recording of CSF drainage is that CSF was drained relatively infrequently.

**Fig 3:**
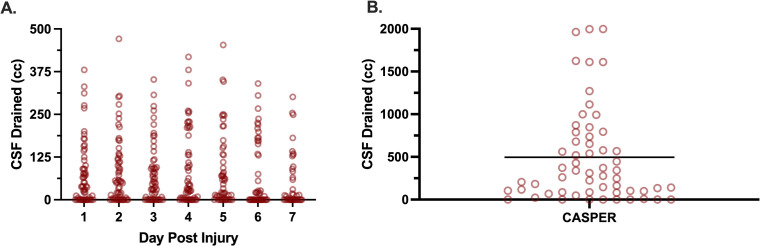
Volume of CSF drained. (**A)** daily and **(B)** total CSF drained. Each symbol represents one participant, horizontal line on Panel represents the median total volume of CSF drained from CASPER participants. *Abbreviations:* CASPER, The Canadian-American Spinal Cord Perfusion and Biomarker Study; CSF, cerebrospinal fluid.

We further examined the instances where some CSF drainage was recorded to determine how much CSF was actually drained. In 21% (536/2597) of observations, less than 5 cc of CSF was drained, in 42%, (1090/2597) between 5 and 10 cc was drained, and in the remaining 37% (971/2597), over 10 cc was drained. When viewing the entire monitoring period, there was a total of 495cc (95% CI [350,641]) of CSF drained, on average (range: 0–1998cc, Fig 3). This equated to an average of 3.37cc/hr (95% CI [2.45, 4,28]) of CSF drained over the entire monitoring period (range: 0–12.36cc/hour). As illustrated in Fig 3, there were no differences between the total volume of CSF drained on any day compared to another, suggesting that the ability to drain CSF did not change over time.

### Hemodynamic Data (MAP, ITP, SCPP)

**Fig 4:**
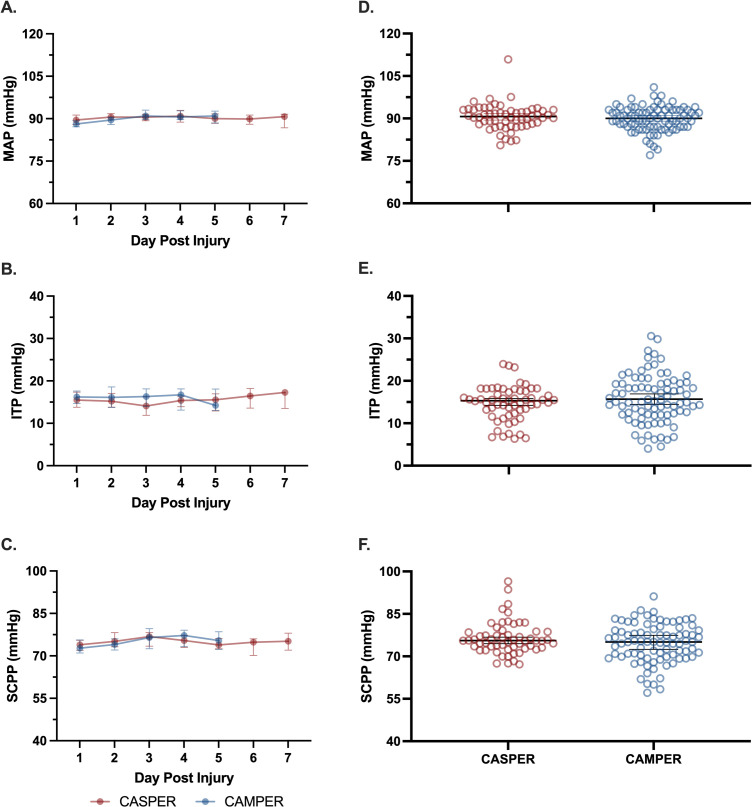
Hemodynamic data comparing CASPER and CAMPER (historical control) studies. Left panels **(A–C)** represent daily median and 95% confidence intervals. Right panels **(D–F)** represent median and 95% confidence intervals over entire monitoring period. Each symbol in D–F represents one participant. There were 136 (95% CI [126,146]) and 90 (95% CI [89,91]) hemodynamic recordings taken in the CASPER and CAMPER trials, respectively. *Abbreviations:* CASPER, the Canadian-American Spinal Cord Perfusion and Biomarker Study; CAMPER, the Canadian Multicentre CSF Pressure and Biomarker Study; ITP, intrathecal pressure; MAP, mean arterial pressure; SCPP, spinal cord perfusion pressure.

**Fig 5:**
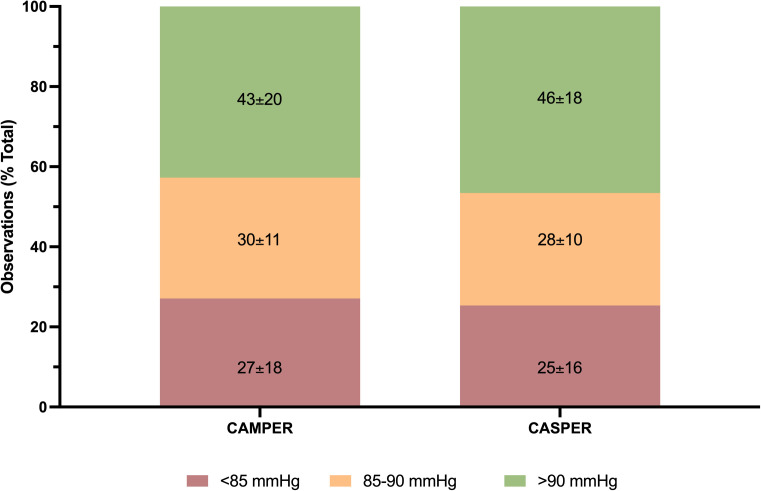
Mean arterial pressure distribution. Mean ± SD percentage of mean arterial pressure recordings *<85, 85–90, and >90 mmHg.* Data represent a total of 7,955 and 8,113 observations in CASPER and CAMPER, respectively. *Abbreviations:* CASPER, the Canadian-American Spinal Cord Perfusion and Biomarker Study; CAMPER, the Canadian Multicentre CSF Pressure and Biomarker Study.

There were 136 (95% CI [126,146]) and 90 (95% CI [89,91]) hemodynamic recordings taken approximately hourly in the CASPER and CAMPER trials, respectively. There were no significant differences between the CASPER and CAMPER participants in individual mean MAP (*d = 0.19, p* = 0.395), mean ITP (*d = 0.20, p* = 0.232), or mean SCPP (*d = 0.28, p* = 0.101) over the entire monitoring period of each study (Fig 4). Similarly, when examining how MAP was maintained relative to the conventional target of 85–90 mmHg, there were no significant differences between the CASPER and CAMPER trials in the distribution of MAP recordings reported as <85 mmHg (25% (95% CI [21,30]) vs. 27% (95% CI [23,31), *d = 0.10, p* = 0.554), 85–90 mmHg (28% (95% CI [25,31]) versus 30% (95% CI [28,33]) %, *d = 0.21, p* = 0.256), or >90 mmHg (47% (95% CI [42,51]) versus 43% (95% CI [39,47]), *d = 0.19, p* = 0.267) (Fig 5). There was greater variability in MAP among participants in whom SCPP was actively managed, rather than MAP (coefficient of variation = 10% (95% CI [9,11]) versus 9% (95% CI [8,9]), *d = 0.43, p* = 0.014). Participants in CASPER managed according to the SCPP target were less prone to have SCPP recordings <65 mmHg as reflected by 1. a lower percentage of total SCPP recordings <65 mmHg than those managed by conventional MAP targets in CAMPER (11% (95% CI [9,13]) versus 16% (95% CI [12,20]), *d = 0.38, p* = 0.029), and 2. a lower cumulative SCPP <65 mmHg based upon an “area under the curve” analysis of SCPP over time (S3 Fig). This is consistent with the study protocol and intention to keep the SCPP ≥65 in the CASPER study.

### Associations between CSF drainage and the change in ITP

**Fig 6:**
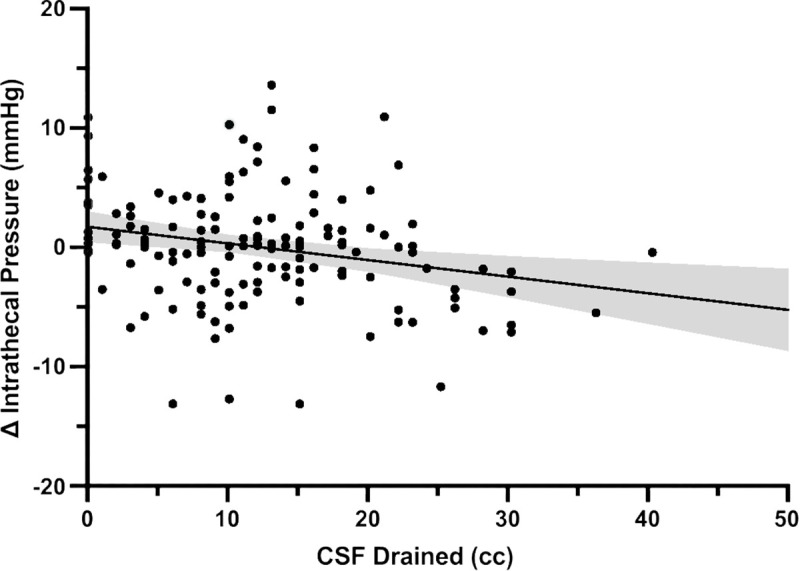
Associations between cerebrospinal fluid drainage and changes in intrathecal pressure. Regression and 95% confidence intervals are shown. Each symbol represents one drainage event (*n* = 145). *Abbreviations:* CSF, cerebrospinal fluid.

This analysis was performed in a subset of six consecutive participants at the UBC/VGH site (100% male, 40 years (95% CI [14,65]), C3-T4), where 24 ′CSF drainage events’ per participant (95% CI [5,44]) were documented to explore associations between CSF drainage and the change in ITP. The mean time for each drainage event was 58 min (95% CI [57,59]) and mean CSF volume drained was 12cc (95% CI [11,14]). There was no significant difference between mean pre- and post-drainage ITP values (18.2 mmHg (95% CI [17.5,19.0]) and 18.1 mmHg (95% CI [17.3,18.8]), *d = 0.03, p* = 0.702) with a mean change in ITP of −0.2 mmHg (95% CI [−0.9,0.6]). The beta-coefficient indicated that for 1mL of CSF drainage ITP would change −0.14 mmHg (95% CI [−0.23,-0.05]*, p* = 0.003) with a y-intercept (i.e., 0mL of CSF drained) equal to 1.55 mmHg (Fig 6).

### Intrathecal pressure waveform morphology

**Fig 7:**
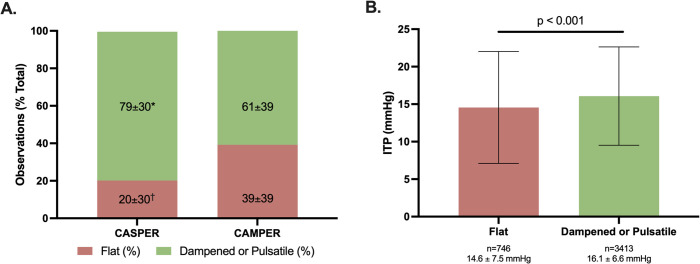
Intrathecal pressure waveform morphology. **(A)** Data are presented as the mean ± SD percentage of waveform observations that were determined by bedside nurses to be flat, dampened, or fully pulsatile. Dampened and fully pulsatile observations are combined. Data represent 8,264 total observations. * indicates *p* = 0.006 vs. CAMPER. ^†^ indicates *p* = 0.021 vs. CAMPER. **(B)** The mean ± SD intrathecal pressure associated with a dampened or fully pulsatile intrathecal waveform morphology was higher than that associated with a flat waveform morphology. *Abbreviations:* CASPER, the Canadian-American Spinal Cord Perfusion and Biomarker Study; CAMPER, the Canadian Multicentre CSF Pressure and Biomarker Study; ITP, intrathecal pressure.

**Fig 8:**
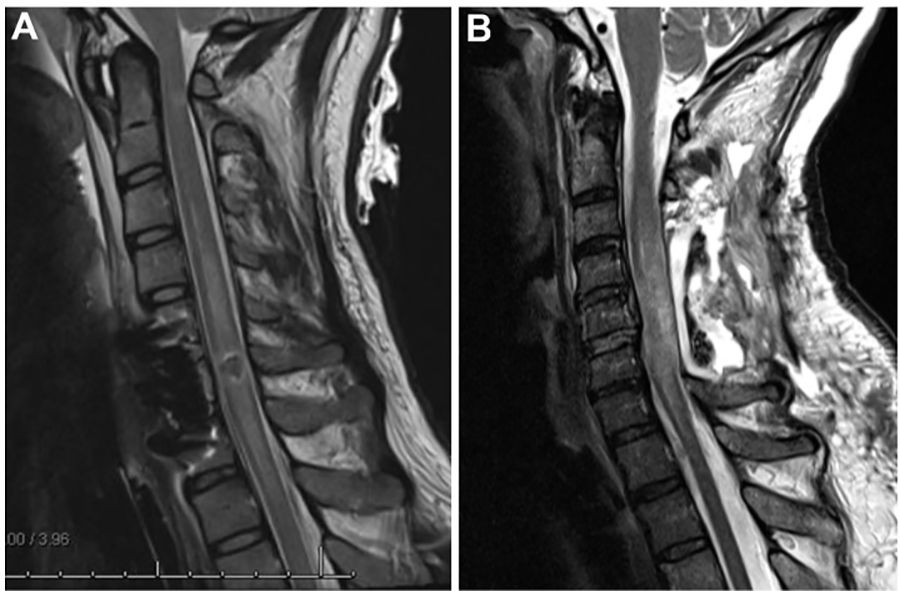
Examples of post-operative MRI to assess patency of SAS after decompression surgery. **(A)** Here, an anterior cervical vertebrectomy of C5 was performed, and the post-operative MRI shows that the SAS is patent around the injury site. In this individual, the ITP waveform that was either dampened pulsatile or pulsatile in 97% of the ITP waveform observations, and a total of 1,998 cc of CSF drained. **(B)** Here, a laminectomy of C3, C4, C5, and C6 was performed, but the post-operative MRI suggests that the SAS is occluded (in this case, the occlusion appears to be caused not only by spinal cord swelling but also posterior epidural fluid/blood). In this individual, the ITP waveform was flat in 97% of the ITP waveform observations, and only 134.5 cc of CSF were drained in the entire monitoring period. *Abbreviations:* CSF, cerebrospinal fluid; ITP, intrathecal pressure; MRI, magnetic resonance imaging; SAS, subarachnoid space.

The ITP waveform was viewed to represent the patency of the SAS around the injury site and was recorded by bedside nurses. As a percentage of all waveform recordings (CASPER = 4,435, CAMPER = 3,829 recordings), the CASPER participants had significantly more waveform recordings noted as dampened or fully pulsatile (79% (95% CI [70,88]) versus 61% (95% CI [52,70]), *d = 0.51, p* = 0.006) and fewer noted as flat (20% (95% CI [11,29]) versus 39% (95% CI [31,48]), *d = 0.53, p* = 0.021) (Fig 7A). An ITP value was recorded at the same time as 4,159 ITP waveform morphology observations. ITP was significantly lower when the intrathecal waveform was deemed to be flat compared to when dampened or fully pulsatile (14.6 mmHg (95% CI [14.0,15.1]) versus 16.1 mmHg (95% CI [15.9,16.3]), *d = 0.23, p* < 0.001; Fig 7B). We did observe cases in which this flat ITP waveform was associated with low ITP and poor ability to drain CSF, in contrast to cases in which a pulsatile ITP was associated with high ITP and the ability to drain CSF easily (Fig 8).

### Vasopressor administration

Participants monitored approximately every hour according to the CASPER protocol were administered vasopressors on fewer observations (% total) than participants enrolled in the CAMPER trial (79% (95% CI [70,88]) versus 96% (95% CI [94,99]) of observations, *d = 0.74, p* = 0.004). Fifty-three of the 58 CASPER participants received norepinephrine during the time they were monitored in the intensive care unit. In addition to norepinephrine, eight participants received vasopressin, one received epinephrine, and one received phenylephrine.

### Adverse events related to CSF drainage

A total of 83 adverse events were reported from the CASPER trial. Thirty-four of 58 participants had at least one adverse event. There were six adverse events reported in CASPER participants that were felt to be probably related to the study intervention (i.e., the lumbar intrathecal drain and the drainage of CSF). There were two documented instances of a “CSF leak at intrathecal catheter site”, two where there was “bleeding at intrathecal catheter site”, and one “intrathecal catheter likely in epidural space (incorrect placement)”. One participant had “CSF positive for bacillus species” in one of the daily CSF specimens sent to the laboratory for microbiological analyses—all CSF samples before and after this were negative, and re-testing this sample was negative, suggesting that this was a contaminant. In no participant was there a diagnosis of bacterial meningitis.

## Discussion

Here, we present the findings of the largest series, that we are aware of, of individuals with acute SCI in whom a protocol for CSF drainage through lumbar intrathecal catheters was implemented to actively manage SCPP. We discovered that consistently implementing such a protocol for CSF drainage across multiple centers with differing (and often long-standing) clinical practices was extremely challenging and in many participants there was little to no CSF that was actually drained over the entire monitoring period. This ultimately resulted in mean hemodynamic measures such as MAP, ITP, and SCPP that were not significantly different in the CASPER cohort than in our historical CAMPER cohort. As such, there were no significant differences in neurologic recovery between CASPER and CAMPER participants. The current study yielded insights about the ITP waveform as a reflection of the SAS at the injury site and revealed a surprisingly weak relationship between the acute drainage of CSF and changes in ITP. These observations highlight considerations for future efforts to develop CSF drainage protocols.

One of the key objectives behind CASPER was to actively maintain SCPP through CSF drainage and then determine if neurologic outcome was improved in those managed according to SCPP targets in comparison to those who were managed with conventional MAP targets. We discovered, however, that it was very challenging to consistently implement the CSF drainage protocol across multiple centers. Ultimately, most of the minute-by-minute decisions about hemodynamic management were made by bedside nurses in the intensive care unit, and while there was an appreciation for maintaining SCPP ≥65 mmHg, it appeared that in many cases this was practically achieved through MAP augmentation rather than ITP reduction. For example, we noted instances where the ITP was > 15 mmHg but the drain was not opened to drain CSF because – *due to a high MAP* – the SCPP was ≥ 65 mmHg and it was therefore deemed that the participant was at the desired SCPP target and did not require intervention. This likely contributed to our finding that CSF drainage occurred relatively infrequently when the drain was in place. At the lead UBC/VGH site, we observed that while the protocol for CSF drainage and SCPP management could be made clear to one bedside nurse, the next day, a different nurse with less advanced knowledge and familiarity with the study could be looking after the participant and revert to a MAP-based management protocol that they were more accustomed to. This required constant (and time-consuming) education at the bedside through the course of the entire seven days of the study and represented a clear obstacle to implementing a consistent protocol within our own institution and undoubtedly at many of the participating centers.

Another contributing factor to the relatively infrequent times that CSF was actually drained in CASPER may also have been related to the ITP waveform. We attempted to use the ITP waveform as a surrogate for the patency of the SAS around the injury site and only drained CSF when the ITP waveform was fully pulsatile or dampened pulsatile and not when it was flat. We did this for two reasons; first, if the ITP waveform is flat and the SAS occluded at the injury site, then it is highly unlikely that opening the CSF drain will achieve much CSF drainage, as there will not be much CSF to drain within the intrathecal space distal to the level of the occlusion. Second, if the SAS is occluded at the injury site, it is possible that a pressure gradient exists across the injury site such that the ITP is different above and below the injury, and ultimately the ITP measured in the lumbar cistern is not representative of the ITP at the injury site. A true assessment of pressure at the injury site would then require a pressure monitor inserted into the SAS right at the injury site, which is the technique employed by Papadopolous and colleagues to measure “intraspinal pressure” when the spinal cord swells to fill the SAS [20]. Other techniques such as contrast-enhanced ultrasound imaging of the spinal cord itself may provide direct perfusion measurements at the injury site [21]. Aside from monitoring the status of the SAS, post-injury monitoring of the spinal cord with serial MRI [22], or with even bedside ultrasound [23], will provide invaluable (and potentially actionable) information about the physiologic condition of the spinal cord at the injury site.

Our observation that ITP was significantly lower with a flat waveform than a fully or dampened pulsatile waveform (see Fig 7B) is consistent with the notion of the SAS being occluded and establishing a pressure gradient across the injury site. That this occlusion of the SAS blocks the transmission of the CSF pulsations and results in the flat ITP waveform in the lumbar cistern makes intuitive sense. This is also consistent with the work of Papadopoulos and colleagues who installed ITP probes at both the site of injury and in the lumbar cistern in a small cohort of individuals with acute SCI and showed that the measurement of pressure in the lumbar cistern can be quite different from what is measured at the injury site [20], presumably when the SAS is occluded at the injury site by the swollen spinal cord. The other important issue around the general hemodynamic management approach that arises from this is that if the SAS is occluded and the resulting ITP is ‘artificially low’ in the lumbar cistern, then subtracting it from MAP will result in a SCPP that is ‘artificially high’ and actually *overestimated*. To address this, we recommended within the CASPER protocol to revert to conventional MAP targets when the ITP waveform was flat.

What our observations about ITP and the ITP waveform morphology highlight is that the hemodynamic management approach of lumbar ITP monitoring and CSF drainage in acute SCI is closely linked to the surgical decompression technique of the injured spinal cord and the achievement of a patent SAS around the injury site. The distinction between a patent and occluded SAS and how this related to ITP waveform pulsatility and CSF drainage is illustrated in Fig 8. Seminal work by Bizhan Aarabi has revealed the importance of the surgical technique in achieving such a patent SAS around the injury site [24]. The findings of Aarabi and colleagues revealed that conventional surgical decompression techniques that include multiple levels of posterior laminectomy are adequate to achieve a patent SAS in the vast majority of cases, and only in a very small number of cases was an aggressive multi-level decompression unable to establish a patent SAS post-operatively and thus potentially rationalize performing an expansile duraplasty.

As these insights were published around the time that we launched CASPER, we did advocate for surgeons to perform multi-level posterior laminectomies as part of the surgical approach to these participants. We believe that the results of the present study indicate that the technique for surgical decompression with an intentional effort to create a patent SAS around the injury site is a critically important component of this hemodynamic management approach of targeting SCPP with lumbar drains and drainage.

Aside from the challenges in implementing the CSF drainage protocol consistently, perhaps one of the most surprising aspects of the CASPER trial was the relationship between CSF drainage and ITP. Fig 6 illustrates that, within a subset of participants, the relationship between the amount of CSF drainage and the resultant change in ITP—at least within the short timeframe of an hour—is quite weak. Given the routine use of CSF drainage in patients undergoing TAAA repair surgery (where the SAS is presumably patent) [25], we had expected a stronger relationship between CSF drainage and changes in ITP in the acute SCI participants within CASPER.

One issue that potentially contributed to this finding is that our ITP threshold of 15 mmHg for CSF drainage was too high, resulting in a drainage protocol that was not aggressive enough to lead to significant changes in ITP. The threshold of 15 mmHg was chosen based on CAMPER data which showed that over two-thirds of the ITPs recorded were over 15 mmHg, and that virtually every participant had ITPs of over 15 mmHg at some point during the study. Intuitively, if the ITP is 17 mmHg and the drain is opened at 15 mmHg, the gradient of only 2 mmHg will lead to very little CSF drainage, and in fact the amount of CSF drained will actually *slow* as the ITP descends towards 15 mmHg. By the same token, in the same setting where the ITP is 17 mmHg and the drain is opened at 15 mmHg, even if 20 cc of CSF were to drain, it would at most change the ITP by 2 mmHg (in essence demonstrating a poor relationship between CSF drained and ITP change similar to what is seen in Fig 6),

We note that in Theodore and colleagues’s (2023) trial of CSF drainage and in past studies of CSF drainage for TAAA repair [26,27], an ITP threshold of 10 mmHg was utilized, and it is certainly possible that setting the ITP lower like this could lead to more CSF actually being drained, and a lower ITP ultimately achieved (with a commensurately higher SCPP) [28]. The Theodore study was considerably smaller than CASPER, with only four participants randomized to receive CSF drainage. However, in this group, the mean ITP was reported to be 5 mmHg; significantly lower than the 15 mmHg observed in individuals following a MAP management protocol. In the study by Theodore and colleagues, at 6-month follow-up, motor scores improved by 57 ± 24 and 15 ± 8 points in the CSF drainage and MAP management groups, respectively. However, the volume of CSF drained, the rate of drainage, the ITP waveform, and the effect of draining a specific volume of CSF on ITP were not reported. Also, the volume of CSF drained (and the drainage rate) that was associated with maintaining ITP below 10 mmHg was not reported, making comparisons with our study difficult. Additionally, it is in some respects challenging to know how the ITP was lowered to 5 mmHg if the CSF drain was set to open at 10 mmHg – one potential reason for this would be an occluded SAS with a pressure gradient across the injury site leading to a very low ITP in the lumbar cistern. Despite these notable protocol differences, the results of this study and our own CASPER trial suggest that in the future a CSF drainage protocol with a lower target ITP is necessary to achieve a desired reduction in ITP.

The “aggressiveness” by which CSF is drained is important to consider in light of the actual normal production and replenishment of CSF. Here, it is worth noting that in the participants where we drained what we consider to be a high volume of CSF that even when draining nearly 2 l of CSF this equates to just over 12 cc/hr of drainage; on average we drained closer to 3 cc/hr. Given the rate of CSF production, it may be that we need to actually achieve quite high volumes of CSF drainage to exceed the natural rate at which CSF is replenished. It is noteworthy that in the setting of TAAA repair surgery where the SAS is patent the rate of CSF drainage is reportedly higher than what we observed, on average, in the SCI participants [25,26].

One of the issues around CSF drainage that raises concerns is the safety of actually doing this. As for the safety of putting lumbar catheters into individuals with acute SCI, we have not observed a high number of complications related to the drain itself, either in CAMPER or in CASPER. In CASPER, we had a few instances of local bleeding or CSF leakage around the skin puncture where the catheter was inserted; these were self-limiting and we did not document anyone with a persistent CSF leak or pseudomeningocele after the drain was removed (as expected for standard lumbar intrathecal catheters). We did not observe neurologic deteriorations in relation to CSF drainage. This is notable given that CSF drainage might worsen a pressure gradient across an occluded SAS which theoretically could worsen neurologic injury. But we also acknowledge that just because acute neurologic deterioration was not observed does not necessarily mean that subsequent potential for neurologic recovery was not affected. Ultimately, we did not observe major differences in neurologic recovery between the CASPER and CAMPER cohorts that suggested CSF drainage (however limited) was associated with reduced neurologic recovery. We did not have any instances of clinical meningitis with the drain in place for either five days (CAMPER) or seven days (CASPER). Based on our data, we would conclude that using CSF drains in the acute SCI population is not associated with significant drain-related adverse events.

One area in which adverse events may theoretically be reduced when employing an SCPP management approach is in the use of vasopressors. Vasopressor use has previously been associated with increased complications in patients with SCI [12,13,29]. Due to differences in institutional protocols and preferences for vasopressor type and administration, we were only able to statistically analyze whether participants did or did not receive a vasopressor on each observation by bedside nurses. Thus, despite this statistically significant reduction in the times that CASPER participants were on vasopressors, it is not possible to definitively conclude that their total vasopressor dose was reduced as compared to the CAMPER participants.

Aside from the limitations that we describe here around the multi-center implementation of the CSF drainage approach for achieving an SCPP of ≥65 mmHg, the low volumes of CSF actually drained, the challenges of SAS patency as reflected by the ITP waveform, and the weak relationship between CSF drainage and ITP, we also observed a fairly high percentage of participants either withdraw or fail to return for follow-up, which of course complicates the interpretation of neurologic recovery at the 6-month mark. Only 43/58 participants included in our analysis returned for 6-month follow-up ISNCSCI exams. However, even if we had achieved 100% follow-up on all participants, it is unlikely that we would have observed differences in neurological recovery compared with participants managed according to the MAP management protocol in CAMPER, given the inherent variability in neurologic recovery.

Now that we have concluded the CASPER study where we found that CSF drainage did not improve SCPP and ultimately had no effect on neurologic outcome, it is reasonable to finally ask *“what then is the role of CSF drainage for the hemodynamic management of acute SCI?”*. It is obvious to us that draining CSF in the acute SCI population is very different (and much more complicated) than draining CSF in the open TAAA surgical repair population where the SAS is patent throughout the spinal axis and one does not need to contend with the issues of spinal cord swelling, SAS patency, surgical decompression technique [24], and the general hemodynamic instability [30] that is often observed in individuals with acute SCI. We believe that lumbar CSF drainage and SCPP management are feasible, but require a thoughtful approach towards surgical decompression, thus linking this form of hemodynamic management to the early surgical management of acute SCI (i.e., the two mainstays of clinical treatment for acute SCI). Thus, the concept of draining CSF to manage SCPP may only be applicable to select acute SCI patients where the SAS is or can be made patent after surgical decompression, at institutions where such patency and CSF drainage can be carefully monitored. The concept of surgically achieving a ‘complete decompression’ that was introduced by Aarabi and colleagues [24] is very important here, as this essentially refers to the achievement of a patent SAS around the injury site. In this regard, we feel that it is critically important to monitor SAS patency in participants from admission, during surgery, and for the first week of management. We suggest a number of tools for monitoring the patency of the SAS: (a) intra-operative [31] and bedside ultrasound [23]; (b) repeat follow-up MRI [22]; and/or (c) observation of the intrathecal waveform morphology [14]. Aarabi and colleagues have pointed out that a multi-level laminectomy may be necessary in the cervical spine to optimize the chances of achieving a patent SAS [24].

Post-operative MRI and intra-operative ultrasound will be important adjuncts to confirming the patency of the SAS around the injury site in future efforts to use this approach to SCPP monitoring/management. Based on our data and the results from Theodore and colleagues, it would seem that setting a lower ITP threshold in order to more aggressively drain CSF and reduce ITP is needed, and it would appear from Theodore’s trial that the 10 mmHg target was met without any major adverse events [28]. Hence, if such a CSF drainage study were to be embarked upon in the future, an ITP target of 10 mmHg or even less could be considered in order to increase the gradient between the measured and target ITP. This acknowledges that if the gradient between the measured and target ITP is small, little to no CSF will be drained, and that small volumes of CSF drainage over an hour result in little to no reduction in ITP, as we observed.

The ITP waveform is likely a reasonable indicator of the patency of the SAS, such that a flat waveform reflects an occluded SAS and signals that the measured ITP may be unreliably low, although we acknowledge that we did not routinely perform post-operative MRIs to assess the patency of the SAS while the intrathecal drain was in. In some respects, given that CSF drainage is known to mitigate ischemic injury in TAAA repair and has been shown to be neuroprotective of the spinal cord, it seems very reasonable to pursue this as a neuroprotective strategy in traumatic SCI. Future effort should be guided by the insights gleaned from the CASPER clinical trial, where we learned a great deal about the challenges of implementing a CSF drainage protocol, the relationship between CSF drainage and ITP, and the need to consider the SAS patency and techniques for achieving it post-operatively.

While the ITP waveform may represent a useful alternative endpoint for any future CSF drainage studies, it would obviously be helpful if additional biomarkers could be established to assist these and other acute SCI studies. Reliance upon neurologic recovery as measured by the ISNCSCI examination is challenging in acute SCI studies given the variability in spontaneous recovery. Additional biomarkers such as CSF and blood biomarkers, MRI (or other imaging) features, and electrophysiologic measurements could be immensely helpful for stratifying such acute SCI study participants objectively, better predicting their outcome, and identifying biological effects from the treatment. The development of such biomarkers is ongoing in our field and will ultimately be welcome additions to the conduct of future trials.

The primary limitation in this study was the inconsistent implementation of the CSF drainage/hemodynamic management protocol across eight North American sites. Extensive measures were taken to ensure the trial was conducted according to the protocol at each site including education of nurses at each site by a research nurse based at the primary site (AT), regular online meetings, and a mid-study in-person meeting of all study site principal investigators and research staff supporting the study. Despite these efforts, when evaluating the hemodynamic data, it was apparent that the intended protocol for CSF drainage was not followed precisely in each participant. This in itself provides important lessons for those interested in using lumbar intrathecal catheters and draining CSF for ITP/SCPP monitoring and management in the acute SCI population. Implementing such a protocol for CSF drainage that regularly adjusts for ITP, SCPP, the ITP waveform, and the volume of CSF drained is definitely a divergence from the routine hemodynamic management practices for acute SCI that are familiar to bedside clinicians and requires considerable training/education.

We acknowledge that the use of a historical cohort comes with limitations due to changes in institutional surgical or acute management strategies and injury characteristics between cohorts. However, we felt that the CAMPER cohort represented a reasonable historical control given that all participants also had lumbar drains (like CASPER) and received conventional MAP management. It should also be noted that we did stop recruitment short of what our targeted sample size was, and so it is possible (albeit unlikely) that we were underpowered in the analysis of neurologic recovery. The investigator team decided to stop enrollment because it was clear that adherence to the protocol was very inconsistent across the sites, resulting in CSF drainage that was fairly minimal (even absent in seven participants), infrequent, and ultimately not effective at reducing ITP and increasing SCPP. Even if we had achieved our target enrollment of 100 participants and had somehow observed an improvement in neurologic recovery, it would have been difficult to attribute it to the hemodynamic management approach employed here, given that MAP, ITP, and SCPP were essentially the same between the CASPER and CAMPER groups (as shown in Fig 4). The failure to observe a difference in neurologic recovery is less likely to be a “power issue”, but rather, the inability of the “intervention” to alter the hemodynamic parameters that we posited were relevant to neurologic recovery. That said, a post-hoc sample size calculation indicated that 475 participants would need to be enrolled in each group to achieve a statistically significant difference in motor score recovery between groups (*d* = 0.182, *α* = 0.05, and *β* = 0.80).

In conclusion, adherence to our protocol for actively targeting a SCPP ≥65 mmHg through CSF drainage in the acute management of traumatic SCI across multiple centers was challenging. We observed relatively little CSF drainage and a modest influence on SCPP, which was not associated with greater neurological recovery than conventional MAP management. This study revealed that while the concept of draining CSF to reduce ITP and increase SCPP is well established in TAAA repair, applying this to acute traumatic SCI is considerably more complicated. Given that CSF drainage has been shown to achieve some degree of neuroprotection of the human spinal cord in TAAA repair, there remains promise for this approach in the hemodynamic management of acute traumatic SCI. However, future CSF drainage protocols for traumatic SCI drainage should determine the appropriate subpopulation for this intervention and likely need to ensure that the surgical decompression techniques achieve a patent SAS at the injury site, and will likely need to drain CSF aggressively to achieve a change in the ITP.

## Supporting information

S1 Fig Flowchart to inform the hemodynamic management of participants enrolled in the CASPER clinical trial. Following the insertion of the lumbar catheter, the first determination sought to intervene with vasopressors if the participant was clearly hypotensive and required resuscitation. But if not hypotensive, the focus was on maintaining an SCPP of 65 mmHg through CSF drainage if the ITP was >15 mmHg. *Abbreviations:* BP, blood pressure; CSF, cerebrospinal fluid; ITP, intrathecal pressure; MAP, mean arterial pressure; SCPP, spinal cord perfusion pressure.
(TIF)

S2 Fig Examples of non-adherence to the CASPER protocol for CSF drainage. These figures are short segments of data where MAP, ITP, and SCPP were recorded by the bedside nurses. **(A)** Here, the ITP (blue) is routinely over 15 mmHg, but rather than opening the lumbar drain-to-drain CSF and trying to reduce the ITP, the MAP (black) was elevated between 96 and 106 mmHg and thus a SCPP of ≥65 mmHg (green) was maintained throughout. **(B)** Here, the ITP is also quite high (ranging from 33 to 43 mmHg), and this time the SCPP transiently drops below 65 mmHg, but again, no attempt was made to drain CSF to reduce the ITP. Examples like this were common and reflected some lack of clarity at the bedside around the CSF drainage protocol, which was intended to drain CSF if the ITP were elevated. *Abbreviations:* CSF, cerebrospinal fluid; ITP, intrathecal pressure; MAP, mean arterial pressure; SCPP, spinal cord perfusion pressure.
(TIF)

S3 Fig Spinal cord perfusion pressure area under the curve. The overall exposure, or “dose” of lower than the intended SCPP of 65 mmHg (measured as the relative SCPP AUC with a threshold set at 65 mmHg) was also lower in CASPER compared to CAMPER participants (0.58 (95% CI [0.44,0.74]) vs. 1.49 (95% CI [1.08,1.91]) mmHg/hour, *d = 0.23, p* < 0.001).
(TIFF)

S1 Table Overview of study sites. (DOCX)

S1 CASPER Protocol Study protocol for lead investigation site. (PDF)

S1 Consort Checklist Completed checklist reporting page on which checklist items are reported in submitted manuscript. This checklist is licensed under the Creative Commons Attribution 4.0 International License (CC BY 4.0; https://creativecommons.org/licenses/by/4.0/).
(DOCX)

S2 Consort Checklist Completed checklist reporting line number on which checklist items are reported in abstract of submitted manuscript. (DOCX)

## Abbreviations

AIS: American Spinal Injury Association Impairment Scale
CAMPER: the Canadian Multicentre CSF Pressure and Biomarker Study
CASPER: the Canadian-American Spinal Cord Perfusion and Biomarker Study
CI: confidence interval
CSF: cerebrospinal fluid
ISNCSCI: International Standards for the Neurological Classification of Spinal Cord Injury
ITP: intrathecal pressure
MAP: mean arterial pressure
SAS: subarachnoid space
SCI: spinal cord injury
SCPP: spinal cord perfusion pressure
TAAA: thoracoabdominal aortic aneurysm
VGH: Vancouver General Hospital
