# Supplementary figures and images for: Targeting spinal cord perfusion pressure in acute spinal cord injury through cerebrospinal fluid drainage: A prospective multi-center clinical trial
Source: PLoS Med. 2026 Feb 5;23(2):e1004925. doi: 10.1371/journal.pmed.1004925 (PMC12890222; doi:10.1371/journal.pmed.1004925)

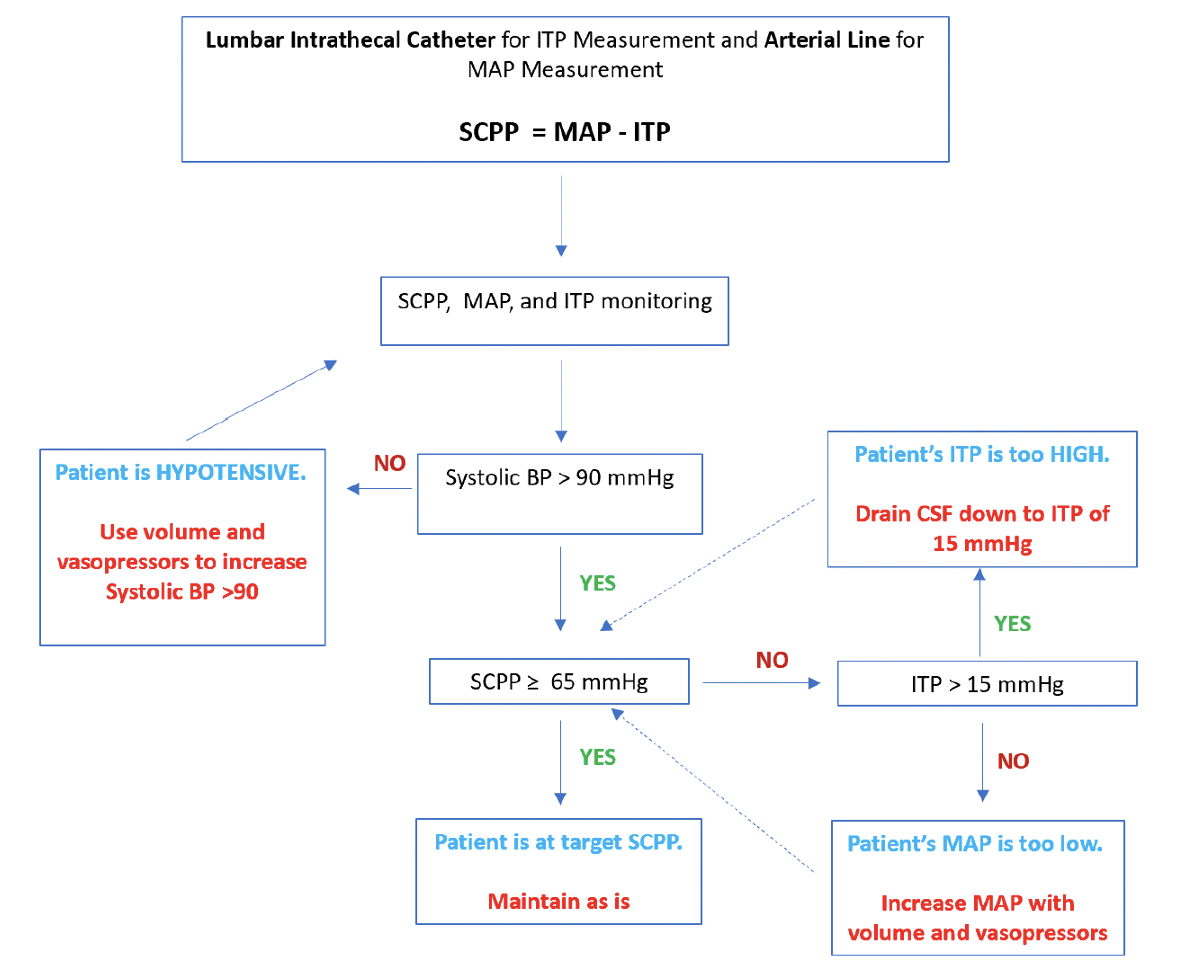

Supplement: S1 Fig — Following the insertion of the lumbar catheter, the first determination sought to intervene with vasopressors if the participant was clearly hypotensive and required resuscitation. But if not hypotensive, the focus was on maintaining an SCPP of 65 mmHg through CSF drainage if the ITP was >15 mmHg. Abbreviations: BP, blood pressure; CSF, cerebrospinal fluid; ITP, intrathecal pressure; MAP, mean arterial pressure; SCPP, spinal cord perfusion pressure. (TIF) [file pmed.1004925.s001.tif]

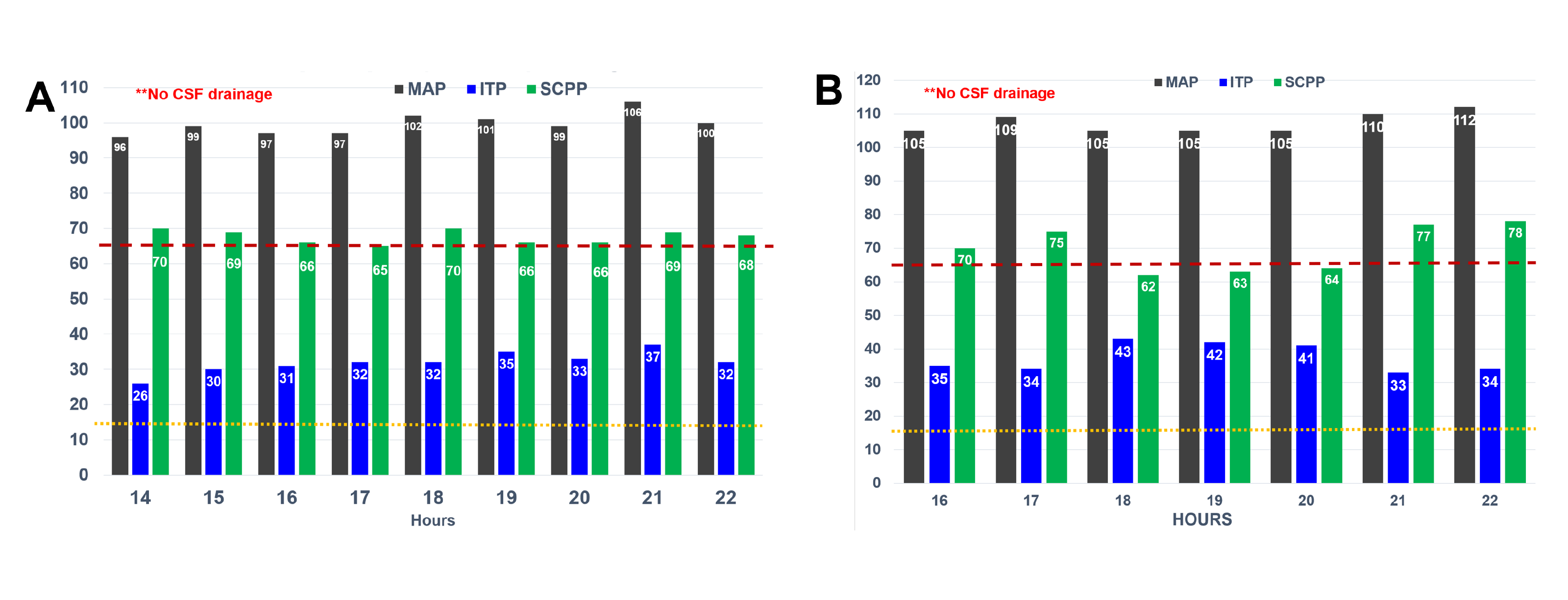

Supplement: S2 Fig — These figures are short segments of data where MAP, ITP, and SCPP were recorded by the bedside nurses. (A) Here, the ITP (blue) is routinely over 15 mmHg, but rather than opening the lumbar drain-to-drain CSF and trying to reduce the ITP, the MAP (black) was elevated between 96 and 106 mmHg and thus a SCPP of ≥65 mmHg (green) was maintained throughout. (B) Here, the ITP is also quite high (ranging from 33 to 43 mmHg), and this time the SCPP transiently drops below 65 mmHg, but again, no attempt was made to drain CSF to reduce the ITP. Examples like this were common and reflected some lack of clarity at the bedside around the CSF drainage protocol, which was intended to drain CSF if the ITP were elevated. Abbreviations: CSF, cerebrospinal fluid; ITP, intrathecal pressure; MAP, mean arterial pressure; SCPP, spinal cord perfusion pressure. (TIF) [file pmed.1004925.s002.tif]

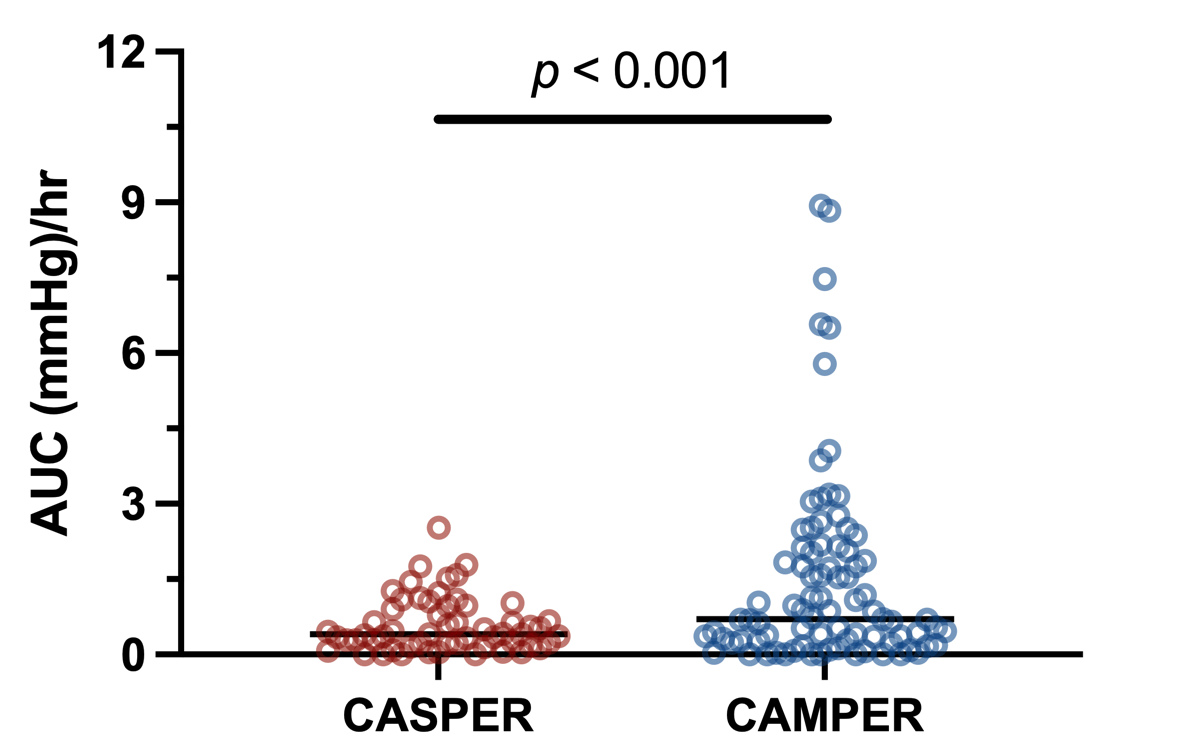

Supplement: S3 Fig — The overall exposure, or “dose” of lower than the intended SCPP of 65 mmHg (measured as the relative SCPP AUC with a threshold set at 65 mmHg) was also lower in CASPER compared to CAMPER participants (0.58 (95% CI [0.44,0.74]) vs. 1.49 (95% CI [1.08,1.91]) mmHg/hour, d = 0.23, p < 0.001). (TIFF) [file pmed.1004925.s003.tiff]
