# Supplementary material for: Targeting spinal cord perfusion pressure in acute spinal cord injury through cerebrospinal fluid drainage: A prospective multi-center clinical trial
Source: PLoS Med. 2026 Feb 5;23(2):e1004925. doi: 10.1371/journal.pmed.1004925 (PMC12890222; doi:10.1371/journal.pmed.1004925)
Supplement: S1 Table — (DOCX) [file pmed.1004925.s004.docx]

**S1 Table.** *Overview of study sites.*

| **Study site (Principal Investigator)** | **Institutional Review Board name (approval number)** |
| --- | --- |
| Vancouver General Hospital (Brian Kwon) | University of British Columbia Clinical Research Ethics Board (H19-00805) |
| Halifax, Queen Elizabeth II Health Sciences Centre (Sean Christie) | Nova Scotia Health Research Ethics Board (1026224) |
| Hôpital Sacré-Coeur de Montréal (Jean-Marc Mac-Thiong) | Centre intégré universitaire de santé et de services sociaux du Nord-de-l'Île-de-Montréal (2021-2278) |
| University of Toronto St. Michael’s Hospital (Jefferson Wilson) | Unity Health Toronto (20-050) |
| UCSF, Zuckerberg San Francisco General Hospital and Trauma Center (Anthony DiGiorgio) | University of California San Francisco (20-32714) |
| University of Nebraska Medical Centre (Jamie Wilson) | University of Nebraska Medical Center (0928-20-FB) |
| University of New Mexico, Health Sciences Center (Christian Ricks) | University of New Mexico Health Sciences Center (21-161) |
| University of Pittsburgh Medical Centre (David Okonkwo) | University of Pittsburgh (STUDY20020031) |
