# Supplementary material for: Targeting spinal cord perfusion pressure in acute spinal cord injury through cerebrospinal fluid drainage: A prospective multi-center clinical trial
Source: PLoS Med. 2026 Feb 5;23(2):e1004925. doi: 10.1371/journal.pmed.1004925 (PMC12890222; doi:10.1371/journal.pmed.1004925)
Supplement: S1 CASPER Protocol — (PDF) [file pmed.1004925.s005.pdf]

STUDY PROTOCOL

"CASPER" – The Canadian-American Spinal Cord Perfusion Pressure and Biomarker Study"

Sponsor: The University of British Columbia

Funders: Praxis Spinal Cord Institute; Paralyzed Veterans of America; Cervical Spine Research Society; Brain Canada; MITACS Inc.

Protocol Version: 2.3

Protocol Number: RHI2019-21

Date: January 17, 2023

ClinicalTrials.gov ID: NCT03911492

Principal Investigator: **Brian K. Kwon,** MD, PhD, FRCSC[1,2,4]
Professor and Canada Research Chair in Spinal Cord Injury
Dvorak Chair in Spine Trauma
Faculty of Medicine, Department of Orthopaedics, UBC
818 West 10th Avenue, Vancouver, BC, V5Z 1M9
Phone: (604) 875-5857 Fax: (604) 875-5858
Brian.Kwon@ubc.ca

Co-investigators:
Marcel F. Dvorak, MD, MBA, FRCSC[1,4]
Charles G. Fisher, MD, MHSc, FRCSC[1,4]
Nicolas Dea, MD, MSc, FRCSC[1,5]
John Street, MD, PhD, FRCS (Tr & Orth)[1,4]
Scott Paquette, MD, FRCSC[1,5]
Raphaële Charest-Morin, MD, FRCSC[1,4]
Tamir Ailon, MD, MPH, FRCSC[1,5]
Lise Belanger, RN, BScN, MSN[6]
Leanna Ritchie, RN[6]
Angela Tsang, RN, BScN[6]
Donald Griesdale, MD, PhD, FRCPC[3]
Mypinder Sekhon, MD, FRCPC[3]
Christopher West, PhD[2]
Sanjay Dhall, MD[7]
David Okonkwo, MD, PhD[8]
Jefferson Wilson, MD, PhD, FRCSC[9]
Charlotte Dandurand, MD, MSc, FRCSC[1,5]

1. Vancouver Spine Surgery Institute, Vancouver General Hospital, Vancouver, BC
2. International Collaboration on Repair Discoveries (ICORD), The University of British Columbia (UBC)
3. Division of Critical Care Medicine, Department of Medicine, UBC
4. Department of Orthopaedics, UBC
5. Division of Neurosurgery, Department of Surgery, UBC
6. Vancouver Spine Program, Vancouver General Hospital, Vancouver, BC
7. Zuckerberg San Francisco General Hospital and Trauma Center, University of California, San Francisco
8. Department of Neurological Surgery, University of Pittsburgh
9. St. Michaels Hospital, University of Toronto

Signatures

By signing this protocol, the Site Principal Investigator agrees to conduct the study in accordance with the protocol, generally accepted standards of good clinical practice, and all applicable federal, state, and local laws, rules, and regulations relating to the conduct of the clinical study.

By signing this protocol, the Study Principal Investigator agrees to be responsible for implementing and maintaining quality control and quality assurance systems with written procedures to ensure that the trials are conducted and data are generated, documented, and reported in compliance with the protocol, accepted standards of good clinical practice, and all applicable federal, state, and local laws, rules, and regulations relating to the conduct of the study.

SITE PRINCIPAL INVESTIGATOR:

____
Signature

____ ________________________
Printed Name Date (DD-MMM-YYYY)

____
Institution

STUDY PRINCIPAL INVESTIGATOR:

____ ________________________
Brian K. Kwon, MD, PhD, FRCSC Date (DD-MMM-YYYY)
The University of British Columbia

TABLE OF CONTENTS

1. STUDY SYNOPSIS 5

2. BACKGROUND INFORMATION / STUDY RATIONALE 6

2.1. Hemodynamic Management of Acute SCI 6
2.2. The Relationship Between MAP and SCPP 7
2.3. The Evaluation of ITP and SCPP in Traumatic SCI 8
2.4. The Canadian Multicenter CSF Pressure and Biomarker Study (CAMPER) 10
2.5. CSF Biomarkers 15

3. RESEARCH OBJECTIVES 17

4. ELIGIBILITY CRITERIA 19

4.1. Inclusion Criteria 19
4.2. Exclusion Criteria 19

5. STUDY PROCEDURES 21

5.1 Enrolment 21
5.2 Lumbar Intrathecal Catheter Insertion 22
5.3 Measurement of Mean Arterial Pressure (MAP), Intrathecal Pressure (ITP), and Active Management of Spinal Cord Perfusion Pressures (SCPP) 23
5.4 CSF and Blood Collection and Processing for Biomarker Studies and Biobank Storage 29

6. MEASUREMENT OF CLINICAL OUTCOMES 32

6.1. International Standards for Neurologic Classification of Spinal Cord Injury (ISNCSCI) 32
6.2. Neuropathic Pain – DN4 Questionnaire and International SCI Pain Basic Dataset 32
6.3. Spinal Cord Independence Measure (SCIM) III 33

7. SAFETY / ADVERSE EVENTS 35

7.1. Defining, Grading and Recording of AEs 35
7.1.1. Definition of an AE & Serious Adverse Event (SAE) 35
7.1.2. AE Grading 36
7.1.3. Recording of AEs 36
7.1.4. SAE Reporting 37
7.2. Relationship Between AEs and CSF Drainage or Insertion, Presence, or Withdrawal of the Lumbar Intrathecal Catheter 37
7.3. Complications Associated with Lumbar Intrathecal Catheter Insertion or CSF Drainage 37
7.3.1. Post-dural Puncture Headache 38
7.3.2. Neurologic Injury from Lumbar Intrathecal Catheter Insertion or CSF drainage 38
7.3.3. Thromboembolic Prophylaxis for Intrathecal Catheter Insertion and Removal 38
7.3.4. Meningitis 39
7.3.5. Additional Note on AEs Related to Intrathecal Catheters 40

8. BIOCHEMICAL OUTCOMES & LONG TERM STORAGE 41

8.1. Central Laboratory & Long Term Storage in the International Spinal Cord Injury Biobank (ISCIB) 41
8.2. CSF Biomarkers for Injury Stratification and Prediction of Outcome 41
8.3. Biospecimen Storage within ISCIB 42

9. DATA COLLECTION AND DATA MANAGEMENT 44

10. EFFICACY ENDPOINT 44

ABBREVIATIONS

**ADL**	**Activities of Daily Living**
**AE**	**Adverse Event**
**AIS**	**ASIA Impairment Scale**
**ASIA**	**American Spinal Injury Association**
**AUC**	**Area Under the Curve**
**CTCAE**	**Common Terminology Criteria for Adverse Events**
**CRF**	**Case Report Form**
**CSF**	**Cerebrospinal Fluid**
**ELISA**	**Enzyme Linked Immunosorbent Assay**
**GCP**	**Good Clinical Practice**
**GCS**	**Glasgow Coma Scale**
**GFAP**	**Glial Fibrillary Acidic Protein**
**GRP**	**Global Research Platform**
**ICF**	**Informed Consent Form**
**ICH**	**International Conference on Harmonization**
**IL-6**	**Interleukin-6**
**IP-10**	**Interferon Gamma-induced Protein 10**
**ISNCSCI**	**International Standards for Neurological Classification of Spinal Cord Injury**
**ITP**	**Intrathecal Pressure**
**MAP**	**Mean Arterial Pressure**
**MCP**	**Monocyte Chemoattractant Protein**
**PAGE**	**Polyacrylamide Gel Electrophoresis**
**PDPH**	**Post-dural Puncture Headache**
**PCC**	**Pearson Correlation Coefficients**
**REB**	**Research Ethics Board**
**Praxis**	**Praxis Spinal Cord Institute**
**ROC**	**Receiver Operating Characteristic**
**SAE**	**Serious Adverse Event**
**SCI**	**Spinal Cord Injury**
**SCISN**	**Spinal Cord Injury Solutions Network**
**SCPP**	**Spinal Cord Perfusion Pressure**
**TNF**	**Tumour Necrosis Factor**
**TNFR-1**	**Tumour Necrosis Factor Receptor-1**
**VGH**	**Vancouver General Hospital**

1. STUDY SYNOPSIS

Currently, the clinical practice guidelines for acute spinal cord injuries (SCI) recommend maintaining mean arterial pressure (MAP) at 85-90 mmHg for 7 days in patients with acute traumatic SCI. Data from the past CAMPER study has indicated that the spinal cord perfusion pressure (SCPP) is more closely related to neurologic recovery than the MAP. In the CAMPER study, SCPP was calculated as the difference between MAP and the intrathecal pressure (ITP) as measured with a lumbar intrathecal catheter.

Hence, the primary research question in this study is, *"Does the active management of SCPP with maintenance at ≥ 65 mmHg result in better neurologic recovery than conventional hemodynamic management which aims solely to maintain the MAP?"*

Specific Aims

This multicenter study will enroll 100 patients with acute traumatic cervical and thoracic SCI who have a lumbar intrathecal catheter inserted within 48 hours of their injury. The lumbar intrathecal catheter will be inserted pre-operatively for the measurement of ITP and the collection of cerebrospinal fluid (CSF) samples. SCPP will be calculated as the difference between MAP and the ITP.

There are two important distinct yet related objectives in this prospective interventional study.

1. Determine the effect of SCPP maintenance ≥ 65 mmHg in acute SCI on neurologic recovery as measured by ASIA Impairment Scale (AIS) grade conversion and motor score improvement.
2. Collect CSF and blood samples for the measurement of neurochemical biomarkers and storage for future biomarker discovery and validation studies.

2. BACKGROUND INFORMATION / STUDY RATIONALE

2.1. Hemodynamic Management of Acute SCI

Currently, for patients who present with acute SCI, there are few available treatment options to potentially improve neurologic outcome. One is **early surgical decompression**. In cervical SCI patients, this may afford some degree of neuroprotection and appears to improve neurologic outcome. The second is **aggressive hemodynamic management** by augmenting MAP to 85-90 mmHg during the first week of injury to ensure that the injured spinal cord receives adequate blood supply.

The importance of aggressive hemodynamic management and the potential for improving neurologic recovery after acute SCI through vigilant MAP maintenance has been acknowledged for many years. Clinical practice guidelines and literature reviews recommend the avoidance of hypotension and the maintenance of MAP at 85-90 mmHg for one week post-injury [1-4]. The guidelines themselves, however, acknowledge the lack of strong evidence to support this MAP target. Recent work by Manley and colleagues at the University of California, San Francisco (UCSF) has demonstrated that improved neurologic recovery can be associated with small increases in MAP during the first 3 to 7 days post-injury [5]. By analyzing almost 1 million MAP measurements of acute SCI patients, the UCSF team observed that the difference in MAP between those patients who improved their ASIA Impairment Scale (AIS) grade and those who did not improve was less than 5 mmHg. **This suggests that efforts to improve spinal cord perfusion by even a few mmHg can result in clinically meaningful neurologic improvements for the acute SCI patient.**

Even though clinical practice guidelines do exist (and most specialized trauma centers likely endeavor to adhere to them), the hemodynamic management of an acute SCI patient is not straightforward. Simply deciding upon a MAP target (e.g. 85-90 mmHg) does not necessarily mean that this MAP is actually achieved. Both we and the UCSF group have shown that acute SCI patients frequently experience episodes of significant hypotension, despite being managed in experienced SCI centers ostensibly adhering to the guidelines [5, 6]. Therefore, it is recognized that even in specialized centers, there is room to improve upon the hemodynamic management of acute SCI patients.

2.2. The Relationship Between MAP and SCPP

The physiologic relationship between perfusion and MAP that holds true in acute traumatic brain injury (TBI) is also applicable to SCI, such that CPP=MAP-ICP in the brain, and SCPP=MAP-ITP in the spinal cord. Of course, because the measurement of ITP is not typically performed in acute SCI, the hemodynamic management in SCI has focused solely on the MAP. Failure to consider the ITP (and thus SCPP) may be an important factor in our inability to demonstrate a strong relationship between a specific MAP target and neurologic outcome in acute traumatic SCI.

In contrast, for ischemic SCI that occurs in the setting of thoraco-abdominal aortic aneurysm (TAAA) repair where the spinal cord's blood supply is inherently vulnerable, the ITP is considered very carefully and is actively reduced by draining CSF in order to improve SCPP. With rates of ischemic SCI estimated to be around 5-7% in both open and endovascular TAAA repair, the drainage of CSF has now become a widely accepted neuroprotective intervention, supported by the most recent guidelines and a systematic review / meta-analysis [7-9]. While the traumatic SCI setting is obviously different from the atraumatic ischemic SCI setting in TAAA repair, the experience with CSF drainage from TAAA repair simply highlights the principle that **active management of SCPP can provide clinically meaningful neuroprotection to the ischemic human spinal cord.**

2.3. The Evaluation of ITP and SCPP in Traumatic SCI

To this end, we initiated a small prospective randomized clinical trial in 2005 to evaluate the safety/feasibility of draining CSF to lower ITP (thus raising SCPP). Intrathecal catheters were inserted pre-operatively, and patients were randomized to CSF drainage to lower the ITP to 10 mmHg (n=12), or no CSF drainage (n=12) [10]. We had initially predicted that ITP would be high pre-operatively and would decrease with decompression. We observed the exact opposite. The ITP upon insertion of the catheter in all patients was approximately 14 mmHg, but after decompression the peak ITP recorded intraoperatively was, on average, almost 9 mmHg higher (p<0.0001) **(Figure 1)**. In the three days post-operatively, the peak ITP recorded in both the CSF drainage group and no-drainage group was approximately 28-30 mmHg (i.e. 14-16 mmHg higher than pre-operatively). **This demonstrated somewhat surprisingly that during the first 3-5 days post-injury, the SCPP in acute SCI patients often decreases due to significant increases in ITP.** These increases in ITP would otherwise go un-noticed without the placement of a lumbar intrathecal catheter. There were no adverse events (meningitis, dural headache, neurologic deterioration) related to the catheters. Neurologic recovery was not improved, although the trial only randomized 24 patients and was not powered to show differences in neurologic recovery.

Figure 1. Changes in Intrathecal Pressure Following Surgical Decompression

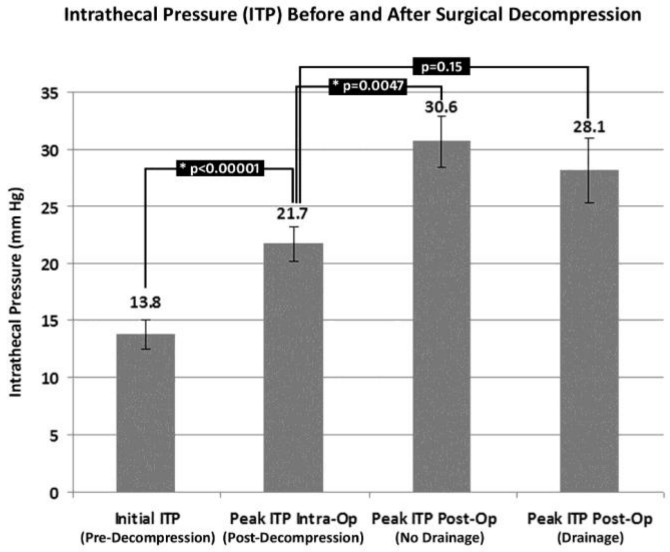

From Kwon et al., J Neurosurg, Spine, 2009

There was an important caveat to the interpretation of our prospective randomized trial of CSF drainage. Because we did not know whether CSF drainage would be safe in acute SCI patients, we were very restrictive in our protocol about how much CSF could be drained over a given time and when such drainage could be instituted. As a result, the entire amount of CSF drained over the full 72 hours in patients randomized to CSF drainage was less than 120 ml, and **a significant reduction in ITP was generally not achieved** [10]. There was a small improvement in SCPP in those randomized to CSF drainage, but this was mainly due to their increased MAP. In essence,

we did not feel that our very restrictive protocol for draining CSF really allowed us to be effective in reducing ITP, but the surprising ITP increases during the post-injury phase suggested that we should continue to at least be monitoring the ITP with intrathecal catheters.

To establish whether changes in MAP and ITP were related and, more specifically, if transient peaks in ITP were associated with similar increases in MAP, the Pearson correlation coefficients (PCC) and the 95% confidence intervals between MAP and ITP were plotted at each hour post-operatively (**Figure 2**). In general, the correlation coefficients are low, and if anything are more frequently negative than positive, suggesting that transient increases in ITP are not associated with increases in MAP that would attenuate the lowering of SCPP. (If anything, the negative PCCs suggest that the MAP is actually dropping when the ITP rises). These findings demonstrate that during the post-operative period, when the ITP transiently rises, the SCPP decreases.

Figure 2. Pearson Correlation Coefficients for Intrathecal Pressure and Mean Arterial Pressure

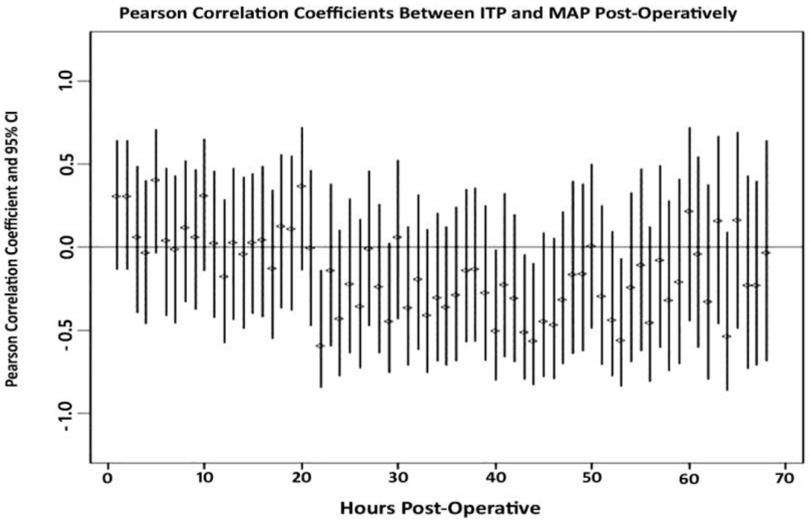

From Kwon et al., J Neurosurg, Spine, 2009

These increases in ITP have important implications for the clinical management of acute SCI. While standard hemodynamic management of acute SCI includes the monitoring and adjustment of MAP, this is typically done without any knowledge of what is happening to the ITP (and hence, the SCPP). Our findings of increased ITP in the intra-operative and post-operative periods indicated that the SCPP was decreased transiently, and these decreases would not be accounted for in the typical clinical management of the acutely injured SCI patient when only the MAP is measured.

This finding therefore formed the basis for further prospective evaluation of ITP and SCPP in acute SCI patients to determine how these physiologic parameters affected neurologic recovery. To this end, we initiated the Canadian Multicenter CSF Pressure and Biomarker Study (CAMPER) (ClinicalTrials.gov Identifier: NCT01279811).

2.4. The Canadian Multicenter CSF Pressure and Biomarker Study (CAMPER)

Following the findings of our small prospective randomized clinical trial on CSF drainage, a decision was made to make the study a prospective observational trial whereby we inserted the lumbar intrathecal catheters but only observed ITP and SCPP (rather than actively draining CSF to reduce ITP). This protocol was initiated locally at Vancouver General Hospital, and then expanded to four other hospitals in: London, Ontario; Halifax, Nova Scotia; Montreal, Quebec; and San Francisco, California.

Inclusion criteria included: cervical or thoracic SCI, admitted within 48 hours of injury, and the ability to obtain a neurologic assessment and informed consent (thus excluding those with head injuries or multi-trauma). The CAMPER protocol involved the insertion of a lumbar intrathecal catheter and the monitoring of ITP for 5 days. No active measures were conducted to change the ITP by draining CSF. CSF and blood samples were obtained approximately 3 times per day for biomarker studies.

In 2017 we reported on the ITP, SCPP, and MAP data and how this related to neurologic outcome. This cohort included 91 patients: 72 males, 19 females, 54 cervical, 37 thoracolumbar, and 57 AIS A, 17 AIS B, and 17 AIS C for whom we had 6 month follow-up data [11].

Firstly, we mapped out every recorded MAP, ITP (or CSF Pressure, "CSFP"), and SCPP for every patient throughout the entire period of time they had the lumbar drain in (**Figure 3**).

Figure 3. MAP, CSFP, and SCPP over 5 days in all patients.

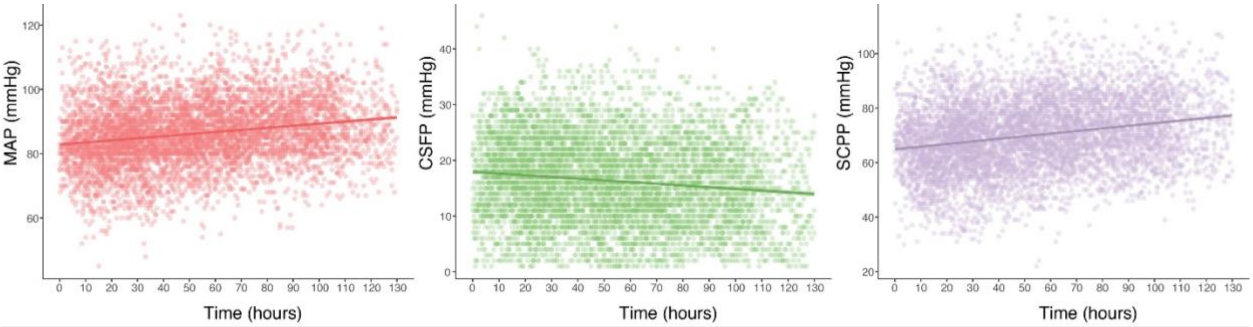

Then, we examined neurologic outcome with respect to AIS grade conversion at 6 months post-injury. We looked at every single recorded MAP, ITP, and SCPP, and examined whether each of these data points was related to AIS grade conversion, or no conversion. This enabled us to generate "transition points" at which point the chance of AIS Grade conversion versus the chance of no AIS Grade conversion was EQUAL (i.e. The 'relative risk' of not improving versus improving an AIS grade was 1.0). For MAP, CSFP, and SCPP these transitions points occurred at 70, 29, and 50 mmHg, respectively (**Figure 4**).

Figure 4. Relative risk of not improving an AIS grade. Here, one can appreciate that the relative risk of not improving an AIS grade increases as the MAP and SCPP decreases, and as the CSFP increases.

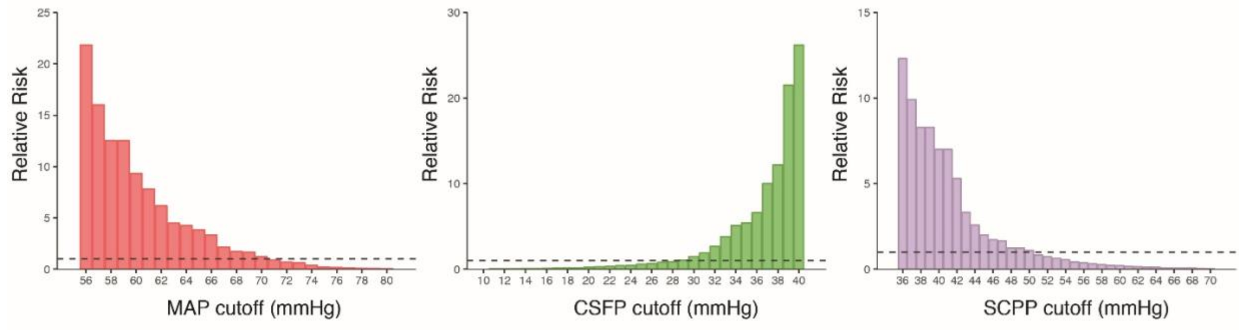

Then, we asked the question of whether suffering episodes of "hypotension" based on decreased MAP below 70 mmHg or episodes of "high pressure" based on increased CSFP above 29, or episodes of "poor perfusion" based on decreased SCPP below 50 was associated with poor neurologic recovery. We found that the number of episodes of low MAP or high CSFP were no different between converters and non-converters. But episodes of low SCPP were much higher in those who did not convert (**Figure 5**). In other words, it was suffering low SCPP (not necessarily low MAP) that portended a poor neurologic outcome. This was true when evaluating all 91 patients (Figure 5A-C) and it was also true when just looking at the 57 AIS A patients (Figure 5D-F). In other words, even in the most severely injured patients (AIS A), neurologic prognosis was still associated with low SCPP.

Figure 5. Episodes of low MAP and high CSFP (ITP) were no different between converters and non-converters, but episodes of low SCPP were much more frequent in the non-converters. This was true for all patients (A-C) and also just the AIS A patients (D-F).

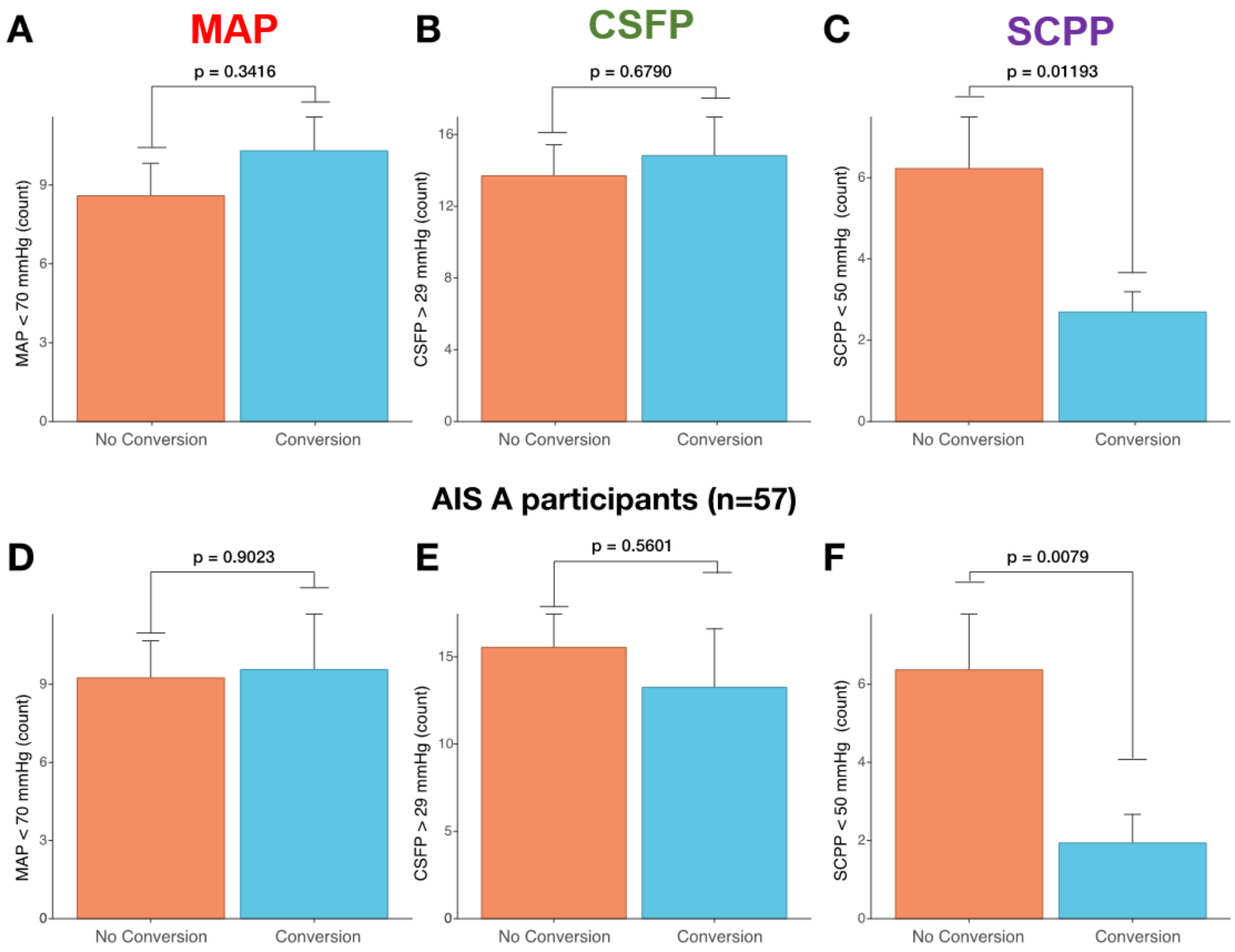

We next looked at how “exposure” to the low SCPP (< 50 mmHg) influenced neurologic recovery based on AIS grade conversion. We found that those who converted and those who did not convert had vastly different “exposures” to low SCPP, particularly in the first 24 hours. In fact, when looking at the entire cohort, exposure to low SCPP in the first 24 hours dropped the chance of conversion by about 50%. This relationship was also true in the AIS A patients. (**Figure 6**)

Figure 6. Exposure to low SCPP is much more common in those who do not convert (i.e. improve) their AIS grade.

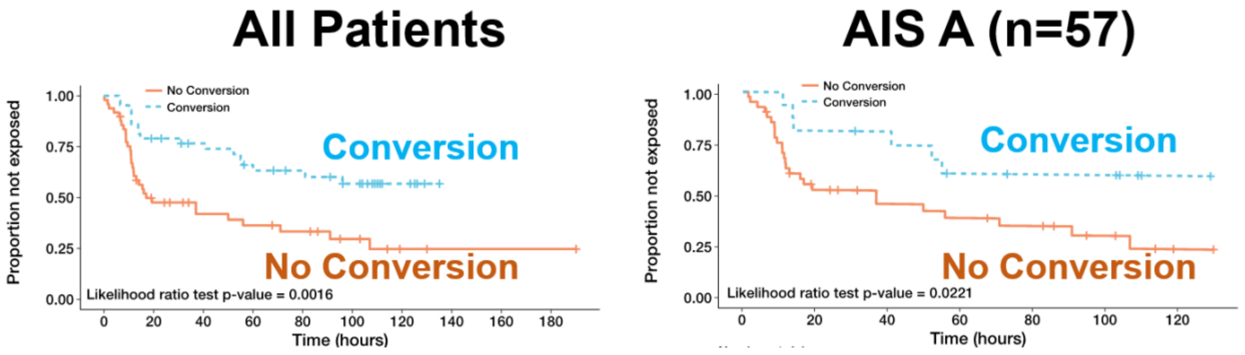

We did a similar analysis to evaluate motor score recovery and asked the question of whether those who achieved motor improvement of 6 or more points were different from those who achieved less than 6 points. Again, we found that exposure to low SCPP was also much higher in those patients who gained less than 6 motor points back (**Figure 7**). This was also true for AIS A patients.

Figure 7. Exposure to low SCPP is much more common in those who do not achieve 6 or more motor points of recovery.

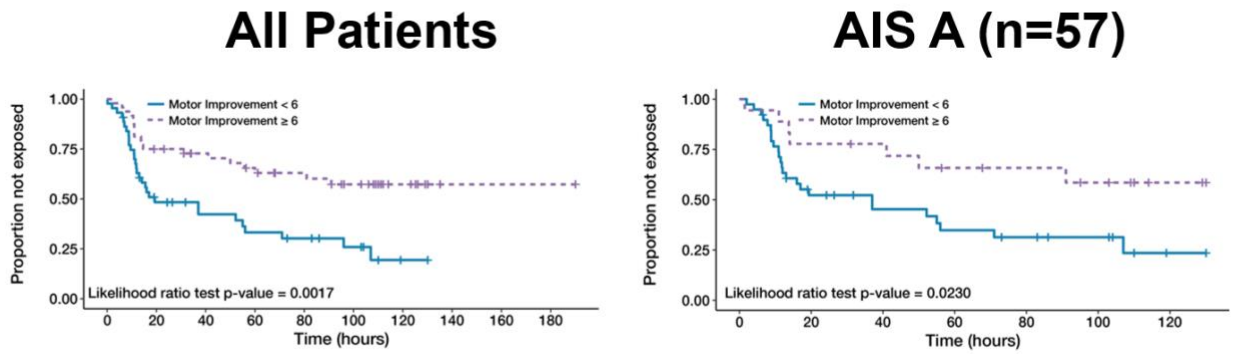

The summary of this data suggested that exposure to low SCPP was associated with a poor neurologic prognosis. Also, it suggested that the SCPP was a more meaningful measure of cord perfusion and was better representative of long-term neurologic prognosis (as compared to the MAP alone – as shown in Figure 5). These findings were published in the journal Neurology, in October 2017 [11]. This 2017 publication of the CAMPER data showed that episodes of low SCPP

(below 50 mmHg) were associated with a poor neurologic prognosis [11]. But what was not addressed was the question of "at what SCPP is the neurologic prognosis optimized?" We have since gone on to assess that with a similar "transition point" analysis where we looked at all SCPPs and evaluated what the risk of "neurologic improvement" was. Here, we see that the relative risk of neurologic improvement is 4-6 times higher with SCPP in the 60-65 mmHg range (**Figure 8**).

Figure 8. Relative Risk of AIS Grade Improvement Based on SCPP. Note that at a SCPP of 65 mmHg, the chances (or "relative risk") of AIS grade improvement are greatest.

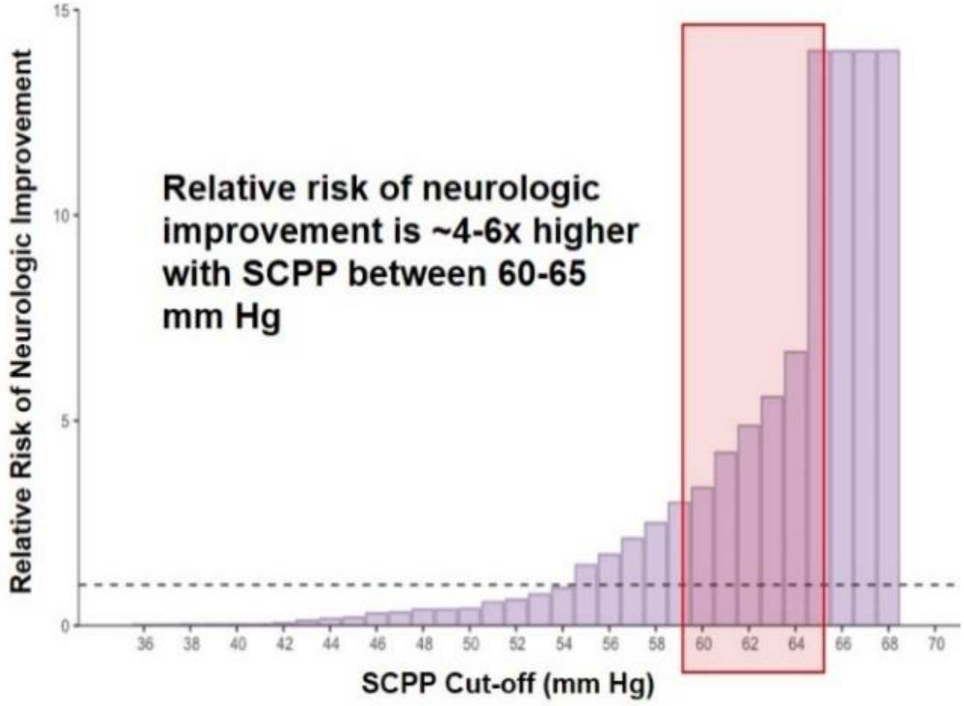

In summary, we have pointed out the importance of hemodynamic management for "squeezing out" every possible gain in neuroprotection for patients with acute SCI. We acknowledge that while guidelines exist for MAP augmentation, they are based on weak evidence. Consistent with the TBI literature, our own data suggests that MAP alone may not be the best reflection of how well the injured neuro-axis is being perfused, and SCPP may be more applicable. We now have data to suggest that actively managing SCPP (to both ensure that it does not drop below 50 mmHg and to strive to achieve a level around 65 mmHg) may improve neurologic outcome. **The purpose of the CASPER study is to now investigate this approach of actively managing SCPP to maintain it at a level of at least 65 mmHg for 7 days (the duration of time generally agreed upon for the management of MAP after acute SCI).**

2.5. CSF Biomarkers

One distinct (albeit closely related) aspect of our CAMPER study was the acquisition of CSF samples to identify neurochemical biomarkers of injury severity. Our interest in biomarkers stems from the fact that spine surgeons are often unable to conduct a baseline neurologic examination on acute SCI patients due to sedation/intoxication or other injuries [12]. Furthermore, even when one can assign a baseline AIS grade, there is much variability in spontaneous neurologic recovery, making it difficult to predict outcome. For example, in the STASCIS study of acute cervical SCI, the AIS grade conversion rate in AIS A patients was 43.2% and 37.0% in early and late decompression groups, respectively [13]. This variability in recovery (and the inability to predict outcome) is a fundamental reason why clinical trials in acute SCI are so difficult to perform and require so many patients (and years) to complete [14].

Our previous work includes a number of studies evaluating CSF as a potential source of objective neurochemical biomarkers of injury severity. This has generated a wealth of information about the pathophysiology of human SCI and has identified potential biomarkers in acute SCI [15-19]. While we are currently conducting expansive proteomic, metabolomic, lipidomic and genomic studies on blood and CSF, we have previously reported that CSF levels of IL-6, IL-8, MCP-1, tau, GFAP, and S100β at 24 hours post-injury were significantly different between AIS A, B, and C groups and could be used to accurately classify baseline injury severity [19] (**Figure 9**).

Figure 9. CSF concentrations of IL-6, IL-8, MCP-1, Tau, S100β, and GFAP are increased according to injury severity at 24 hours post-injury.

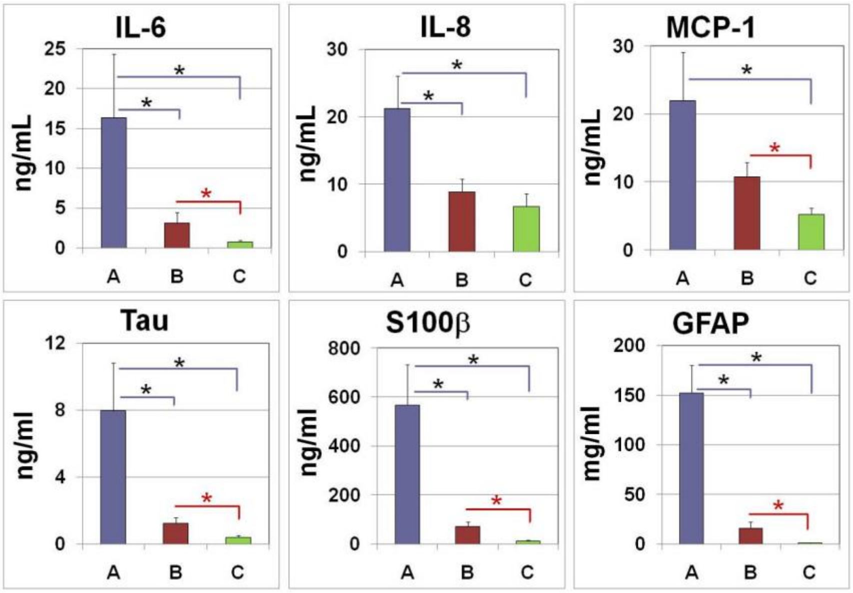

From Kwon et al., J Neurotrauma, 2010

We subsequently reported that these proteins were also different between those who experienced AIS grade conversion and those who did not (**Figure 10**), and could also be used in combination to predict neurologic outcome [16]. In fact, using Linear Discriminant Analysis modeling, we showed that these 6 biomarkers could predict AIS grade conversion in cervical SCI patients with an accuracy of 86.7% (92.9% accuracy at predicting who would NOT convert) [16]. We also showed recently that in cervical SCI, these CSF biomarkers were far better at classifying injury severity and predicting outcome than MRI measures of the injured cord [20]. In fact, when combining CSF biomarkers, MRI biomarkers, and baseline AIS grade in a logistic regression model to predict neurologic recovery, MRI biomarkers and even the knowledge of the patients' baseline AIS grade did not add anything to the model – *the CSF biomarkers were the only significant variables for predicting outcome.*

Figure 10. CSF biomarkers are significantly higher in those who do not convert their AIS grade (NO) as compared to those who do (YES). The y-axis is a log 10 scale, so that a 1-point difference is a 10x difference in concentration. Modified from Kwon et al. J Neurotrauma, 2017.

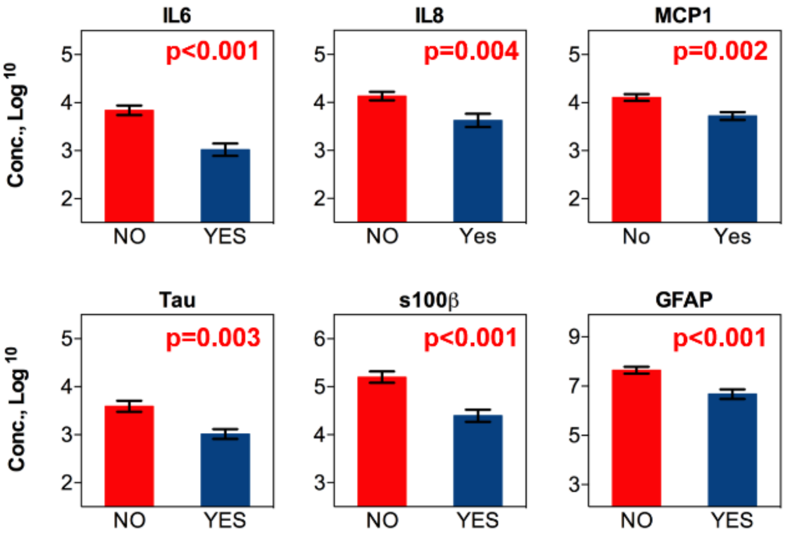

Because we will insert lumbar intrathecal catheters to manage SCPP in this proposal, we can collect CSF samples for the measurement of these 6 CSF biomarkers. **Hence, in this proposal, we will utilize these CSF biomarkers to additionally control for the severity of neurologic impairment and predict recovery.** This will help us in interpreting the true effect of SCPP management on neurologic recovery. For example, if CSF biomarkers from the AIS A patients indicate that they have a 92.9% likelihood of remaining AIS A, and yet AIS grade improvement does occur with SCPP management, then the biomarkers can help us to distinguish this recovery from the variability of spontaneous recovery. It should be noted that while these 6 CSF proteins (IL-6, IL-8, MCP-1, tau, GFAP, and S100β) represent just a handful of the many potential biomarker candidates, they are the proteins that we have studied most extensively and therefore understand the best.

3. RESEARCH OBJECTIVES

Objective 1. To determine if actively maintaining an SCPP of at least 65 mmHg with a combination of MAP augmentation and CSF drainage promotes better neurologic recovery than routine hemodynamic management that focuses solely on MAP augmentation.

We will compare the neurologic recovery in our patients who receive active SCPP management against our prospectively collected CAMPER cohort, a cohort who had intrathecal catheters placed but only routine MAP management. Active SCPP management will be achieved clinically by a combination of MAP augmentation and CSF drainage to lower ITP.

Objective 2. To determine if actively maintaining an SCPP of at least 65 mmHg with a combination of MAP augmentation and CSF drainage will allow for a reduction in the usage of vasopressors in acute SCI.

Data that has emerged from UCSF has really highlighted the potential adverse events (AEs), such as arrhythmias and troponin elevations, related to MAP augmentation with vasopressors [21, 22]. We have also witnessed cardiac ischemia in elderly individuals where vasopressors were aggressively used to augment MAP. In many past cases, we have felt that the acute SCI patient has required extremely high doses of vasopressors to achieve a standard MAP target, and that this could be quite deleterious. So, there definitely is an underlying rationale for determining whether a different approach (one that involves both MAP augmentation and CSF drainage) can reduce vasopressor requirements.

We will be tracking MAP, ITP, and SCPP, and the concomitant vasopressors required to maintain an SCPP of $\geq$ 65 mmHg. We will be able to compare this with vasopressor usage in our past CAMPER patient cohort to determine if this approach can allow for a reduction in vasopressor usage.

Objective 3. To determine the feasibility of draining CSF to reduce ITP in the acute post-injury setting, when the cord may be swollen against the dura causing subarachnoid space occlusion at the injury site.

One observation from our first study of CSF drainage published in 2009 was that the pressure waveform was dampened in patients where the spinal cord was swollen against the dura, occluding the subarachnoid space [10]. We proposed that there was a pressure gradient established across the injury site in such circumstances, where the pressure at and above the injury site might be different than that measured below. Since then, there has been much ground-breaking work from Marios Papadopoulos who has confirmed this phenomenon of subarachnoid space occlusion by sliding a Codman pressure probe into the subarachnoid space between the dura and the swollen spinal cord, right at the injury site [23]. This raises the possibility that it may not be feasible to drain CSF or even measure the ITP at the injury site when the cord is swollen against the dura. We will therefore assess the pressure waveform over time

and determine if CSF drainage for SCPP management is even feasible if the pressure waveform is dampened (implying subarachnoid space occlusion).

Based on our experience, there is considerable variability in how much (and for how long) the cord swells, and how this affects the intrathecal pressure waveform. We also recognize that the spinal cord morphometry differs amongst patients, with some patients having a very large amount of CSF around their spinal cord and others having a very small amount of CSF around the cord. This difference in morphometry and – in essence – the native amount of "room" around the spinal cord will most likely influence whether the swollen spinal cord will be compressed by the dura. In Aarabi et al.'s MRI analysis of acute SCI patients after surgical decompression (published in Dec, 2018), they found that only 11% of patients had the combination of post-injury swelling and native cord morphometry to cause the cord to be swollen up against the dura even after a laminectomy [24]. Our published work from 2017 [11] is consistent with this, that despite the swelling that may occur at the SCI site, the lumbar intrathecal catheter still provides meaningful information about SCPP and has the advantage of technical familiarity with insertion of a lumbar intrathecal catheter without the potential hazards of inserting a catheter into the subarachnoid space right at the site of spinal cord injury.

Objective 4. To determine if there are complications associated with the installation of the intrathecal catheter and drainage of CSF in the acute SCI patient.

It goes without saying that we will measure AEs associated with intrathecal catheter placement. Of course, acute SCI patients incur a huge number of AEs; but we have utilized a system of daily monitoring those that are most applicable to the lumbar catheters – namely CSF leak/postural headache, nausea/vomiting, and infection. The patients will be carefully monitored by the study research team throughout the time that they have the intrathecal catheter placed.

4. ELIGIBILITY CRITERIA

4.1. Inclusion Criteria

Patients who meet the following inclusion criteria are eligible for admission into the study:

- Lumbar intrathecal catheter to be inserted as part of clinical hemodynamic management and CSF sample collected **within 48 hours of injury**
- Initial blood sample collected **within 24 hours of injury**
- Male or Female ≥ 17 (or the provincial age of majority – depending on local REB guidelines)
- Complete (AIS A) or incomplete (AIS B, C) acute traumatic spinal cord injury
- Bony spinal levels between C0 and T12 inclusive
- Blunt (non-penetrating) spinal cord injury treated either surgically or non-surgically
- Able to communicate in English, or with a translator where available, and provide informed consent

4.2. Exclusion Criteria

Patients who fulfill any of the following criteria are not eligible for admission into the study:

- Motor incomplete spinal cord injury **AIS D** (i.e. at least half (half or more) of the key muscle functions below the neurological level of injury (NLI) have a muscle grade greater than or equal to 3/5)
- Spinal cord injury with sensory deficit only (i.e. no motor deficit)
- Isolated radiculopathy
- Isolated cauda equina injury or injury **below** bony spinal level **T12**
- Associated injury (soft tissue or bony) to the lumbar spine where the intrathecal catheter would be placed
- Associated traumatic conditions that would interfere with the outcome assessment (e.g., traumatic brain injury, chest, pelvis, abdomen, or femur injury requiring operative intervention)
- Pre-existing neurodegenerative disorder, such as Parkinson’s disease, Alzheimer’s disease, Huntington’s disease, multiple sclerosis, amyotrophic lateral sclerosis
- Pre-existing thromboembolic disease or coagulopathy, such as hemophilia, von Willebrand disease
- Presence of systemic disease that might interfere with patient safety, compliance or evaluation of the condition under study (e.g., clinically significant cardiac disease, HIV, Hep B or C) HTLV-1
- Pre-existing inflammatory or autoimmune disorder (e.g. rheumatoid arthritis, systemic lupus, psoriasis, or ankylosing spondylitis)
- Any other medical condition that in the investigator’s opinion would render the protocol procedures dangerous or impair the ability of the patient to receive protocol therapy

- Female patients who are pregnant

5. STUDY PROCEDURES

5.1 Enrolment

Spinal cord injured patients will arrive at their respective trauma centers and, after being triaged and stabilized according to standard trauma protocols, the patients will be evaluated by staff (residents or fellows), and a complete neurologic examination will be performed using the International Standards for Neurologic Classification of Spinal Cord Injury (ISNCSCI) examination. The motor-sensory examination will be recorded and the AIS grade will be assigned.

SCI patients will be approached for enrolment in this study ***if the attending spine surgeon has decided that the insertion of a lumbar intrathecal catheter for monitoring ITP and maintaining SCPP is clinically indicated for their hemodynamic management.*** It is important to point out here that the decision to insert the lumbar intrathecal catheter for ITP monitoring and management is a clinical decision made by the surgical team. The rationale for the clinical decision to install a lumbar catheter is derived from our previous publication showing that SCPP is a more meaningful reflection of neurologic recovery than MAP [11]. Centers participating in this study are centers where the spine surgeons have adopted the insertion of the lumbar intrathecal catheter as part of the hemodynamic management of the acute SCI patient. This research protocol will seek to characterize how such active SCPP management influences neurologic outcome.

If the surgeon has decided to insert the intrathecal catheter and actively manage SCPP in the acute SCI patient, the patient will be approached to consider participating in the study. The potential research participant will be informed that the purposes of the study are to 1. evaluate their neurologic recovery over time, 2. obtain CSF and blood samples for biomarker studies, 3. obtain CSF and blood samples for storage in the International Spinal Cord Injury Biobank (ISCIB).

Consent. Consent to participate in the study will be obtained from either **1.** the acute SCI patient himself/herself, or **2.** his/her legally authorized representative (**LAR**). In situations where a patient is able to provide a reliable neurologic assessment but cannot provide informed consent, the LAR will be approached for consent. This situation arises when a patient is able to cooperate and participate in a neurologic examination (and hence a baseline AIS grade and motor score can be obtained) but then in the subsequent hours becomes sedated with analgesics or requires intubation, thus making it difficult to obtain the informed consent directly from the patient.

In addition, patients who consent to participate in the CASPER study in Canada will be informed that they may be subsequently invited to participate in other clinical SCI research studies which are also sponsored/funded by the Praxis Spinal Cord Institute. If they consent to participate in any such studies, participants will be given the option to allow their de-identified data from the CASPER study to be linked with the data from these subsequent Praxis sponsored/funded studies (e.g. the Rick Hansen Spinal Cord Injury Registry) for permitted research purposes. Additional ethical approval will be sought where necessary.

5.2 Lumbar Intrathecal Catheter Insertion

The technique for inserting the lumbar intrathecal catheter will be left to the discretion of the surgical team. In general, the standard technique for catheter insertion is with the patient logrolled into the lateral decubitus position with a clinician supporting the cervical spine if there is an unstable cervical spine fracture. After sterile preparation of the skin, the needle is advanced between L2/3, L3/4, or L4/5 (i.e. in the lumbar spine) to puncture the dura, and once CSF is detected, the intrathecal catheter inserted through the needle and advanced 10-15 cm through the dura.

Individual trauma centers will have their own equipment and protocols for lumbar intrathecal catheter placement. ***Given that the insertion of the lumbar intrathecal catheter is a clinical decision made by the surgical team, the local center should utilize the lumbar intrathecal catheter that is available/familiar to them.***

5.3 Measurement of Mean Arterial Pressure (MAP), Intrathecal Pressure (ITP), and Active Management of Spinal Cord Perfusion Pressures (SCPP)

Based on the CAMPER study, we reported that SCPP as calculated as the difference between MAP and ITP (Intrathecal Pressure measured via a lumbar catheter) was potentially more representative of neurologic impairment than the MAP alone. The proposal within CASPER is therefore to ask the question *"does actively maintaining SCPP at a specific target improve neurologic outcome as compared to standard MAP management alone?"*

Collaborating sites (e.g. UCSF [Dhall] and Univ. of Pittsburgh [Okonkwo]) have already instituted the insertion of a lumbar catheter to measure ITP and calculate SCPP as part of the clinical hemodynamic management strategy for the acute SCI patient. We will take advantage of this clinical practice to address the primary research question of whether active SCPP management is better than conventional MAP management in terms of neurologic recovery. The CASPER study will formally evaluate active SCPP management by tracking neurologic outcomes in patients who are having lumbar intrathecal drains inserted to measure ITP as part of their clinical care. **It is therefore important to have a commonly agreed upon protocol for SCPP management that is adhered to across all sites.**

<u>MAP MANAGEMENT</u>

MAP will be monitored with an arterial line. The focus in the CASPER trial will be on the SCPP, but MAP augmentation, when needed, will be achieved with volume replacement (i.e. crystalloid, colloid, or whole blood) followed by vasopressors as needed. At Vancouver General Hospital, norepinephrine is the vasopressor of choice, based on locally conducted animal and clinical studies [25, 26]. It is recognized however, that other sites may use different vasopressors based on clinical preference/familiarity and that strong clinical evidence to support one vasopressor over another is not available.

What will the MAP target be? Patients should not be allowed to be HYPOTENSIVE and thus, MAP augmentation should be instituted if their systolic BP is less than 90 mmHg. Based on the clinical practice of the specific institution, if the ICU staff wish to maintain MAP at 85 mmHg, this would be accepted. <u>But we will not mandate a target MAP of 85 mmHg</u>.

SCPP MANAGEMENT

SCPP will be calculated as the difference between MAP and the intrathecal pressure (ITP). SCPP will be monitored and displayed in real time on the GE bedside monitor. Based on our CAMPER data, **we will actively maintain SCPP at a threshold of at least 65 mmHg.** While we showed a 4-8x chance of improvement with an SCPP of 60-65 mmHg, here we choose a minimum threshold of 65 mmHg (rather than 60 mmHg) because we recognize that during the course of managing such acute SCI patients, even when adhering to practice guidelines there is inevitably some variation in their hemodynamic parameters [6]. We would rather aim to keep the SCPP above 65 mmHg (and have occasional instances where the SCPP dips beneath it) than set the threshold SCPP lower (e.g. 60 mmHg) and have patients occasionally dropping below this.

ITP MANAGEMENT

The lumbar intrathecal catheter will be connected to a Duet External Drainage and Monitoring System (Medtronic, MN) with the pressure transducer zeroed at the phlebostatic axis. The phlebostatic axis is estimated by drawing an imaginary line from the fourth intercostal space at the sternum and finding its intersection with an imaginary line drawn down the center of the chest below the axillae.

SCPP can be raised by either increasing MAP or decreasing ITP. A key hemodynamic management strategy in CASPER is to decrease ITP so as to not require increasing doses of vasopressor to keep the MAP elevated. Certainly, we have observed cases where significant doses of vasopressor are required to keep the MAP elevated to 85-90 mmHg. If this is done with persistently high ITP, it is possible that the desired SCPP would not be achieved anyways.

What should the ITP target be?

In the CAMPER dataset, if all of the ITP measurements in all patients across all time points were considered, it was noted that 51.4% were above 15 mmHg, and 28.3% were above 20 mmHg (see **Figure 13A**). The graph in **Figure 13B** considers the ITP data on a patient-by-patient basis (each patient represented by a dot, with the median shown by the bar) and addresses the question *"for each patient, what percent of their ITP measurements were above 5, 10, 15, 20, 25, or 30 mmHg?"*. As shown in Figure 13B, for the ITP cut-off of 20 mmHg (yellow), 50% of the patients had ~25% of their ITP measurements greater than 20 mmHg. For an ITP cut-off of 15 mmHg (green), 50% of the patients had ~50% of their ITP measurements greater than 15 mmHg.

Figure 13. ITP measurements in all CAMPER patients (n=92). In 13A, all ITP measurements are plotted in all patients. Note that about 50% are above 15, and 28% are above 20. In 13B, each patient is represented by a dot, and the percentage of each patient's ITP measurements that were above the "ITP-cutoff" is plotted. Note that fewer than 10 patients have 0% of their ITP below that threshold of 15 (green) or 20 mmHg (yellow).

Figure 13A

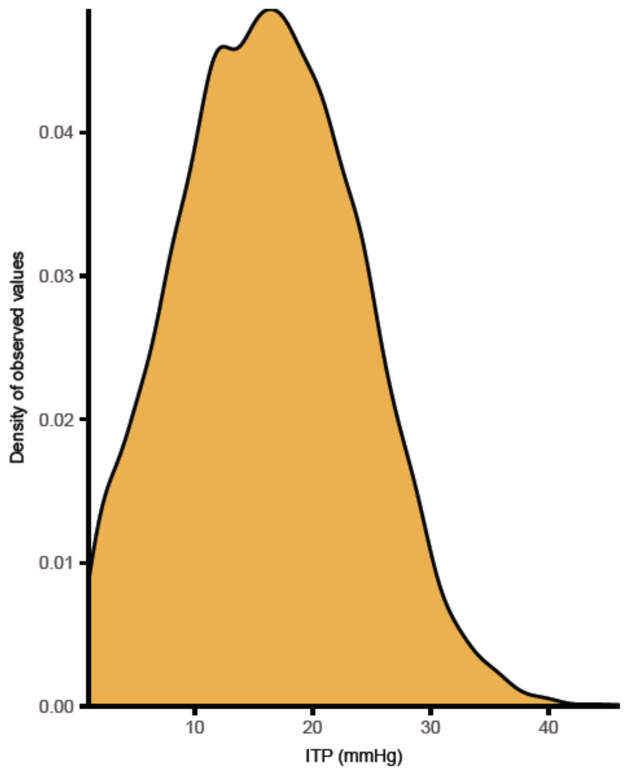

Figure 13B

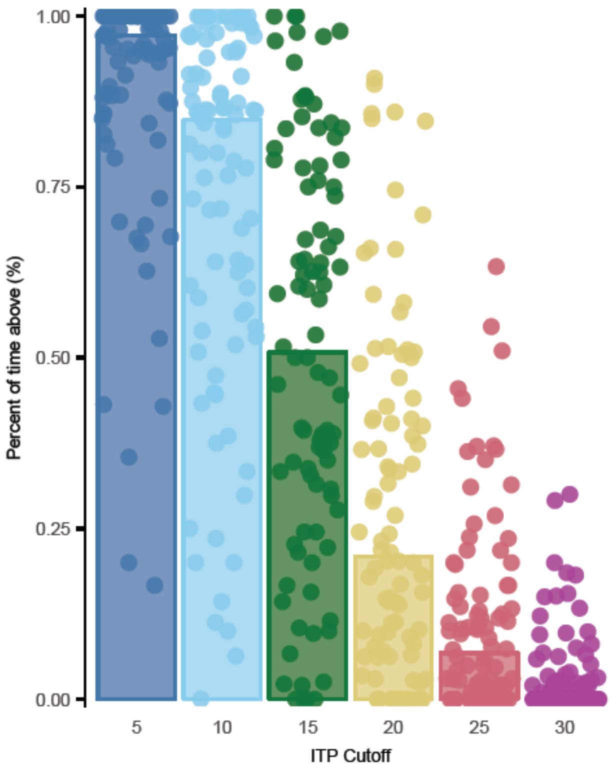

In the analysis of the CAMPER dataset that followed the original Squair et al. paper from 2017, we examined the association between ITP and AIS conversion (**Figure 14**) and found that an ITP threshold of 20 mmHg was associated with improved AIS conversion (*Squair et al, in review*).

Figure 14. Relationship between ITP and AIS Conversion. Here, we plotted the relative risk of improving AIS grade according to ITP. The "risk of improving an AIS grade" was 1.0 at an ITP of 26 mmHg, and then improved with the lowering of ITP to below 20 mmHg. Note that even when the ITP drops below 20 mmHg, the relative risk of neurologic improvement continues to increase. Therefore, keeping the ITP at around 15 mmHg would likely avoid ITP from rising up to 26 mmHg which is the threshold where AIS conversion is not "more likely".

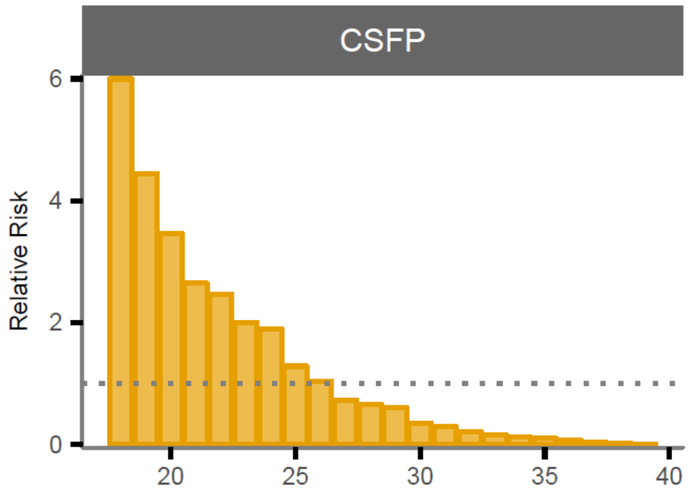

We also looked at the relationship between ITP and motor score recovery (**Figure 15**). With increasing motor score recovery, we found that the mean arterial pressure transition point remained relatively constant (black rectangle regions), while the cerebrospinal fluid pressure (CSFP, or "ITP") and spinal cord perfusion pressure (SCPP) transition points continued to change. Note that as CSFP moves down towards 20, there is greater motor score improvement.

Figure 15. Relationship between ITP and motor score recovery.

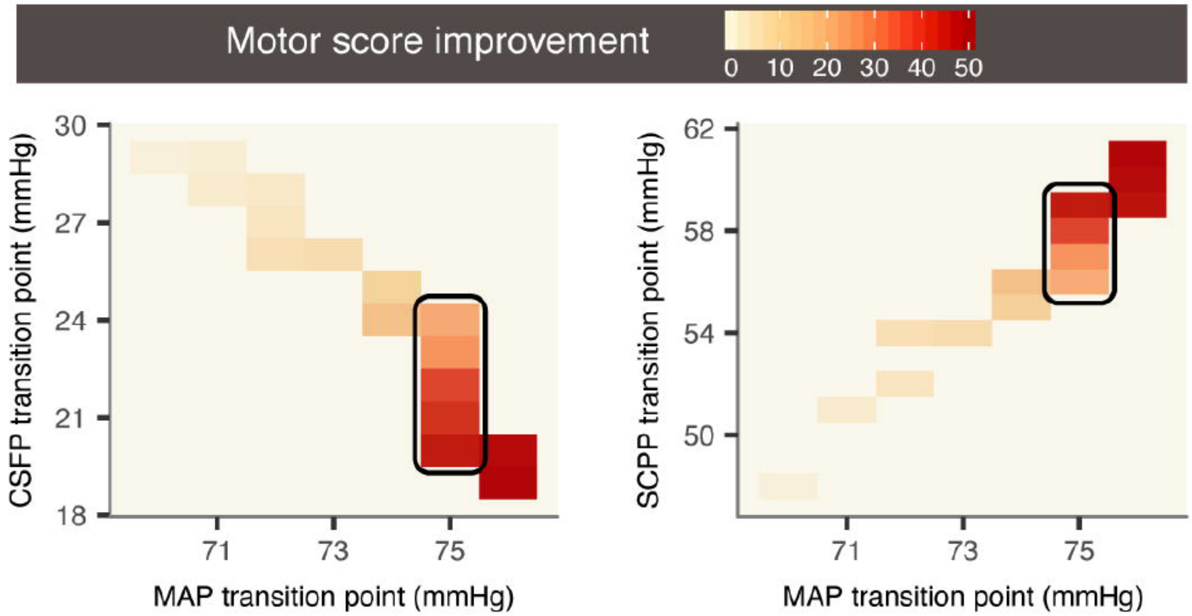

For these reasons, it would seem rational to actively maintain an SCPP target by first limiting the ITP to 20 mmHg with CSF drainage. We would propose here that if the ITP were > 15 mmHg, CSF would be drained to bring it down to 15 mmHg.

Figure 16. Algorithm for SCPP Management

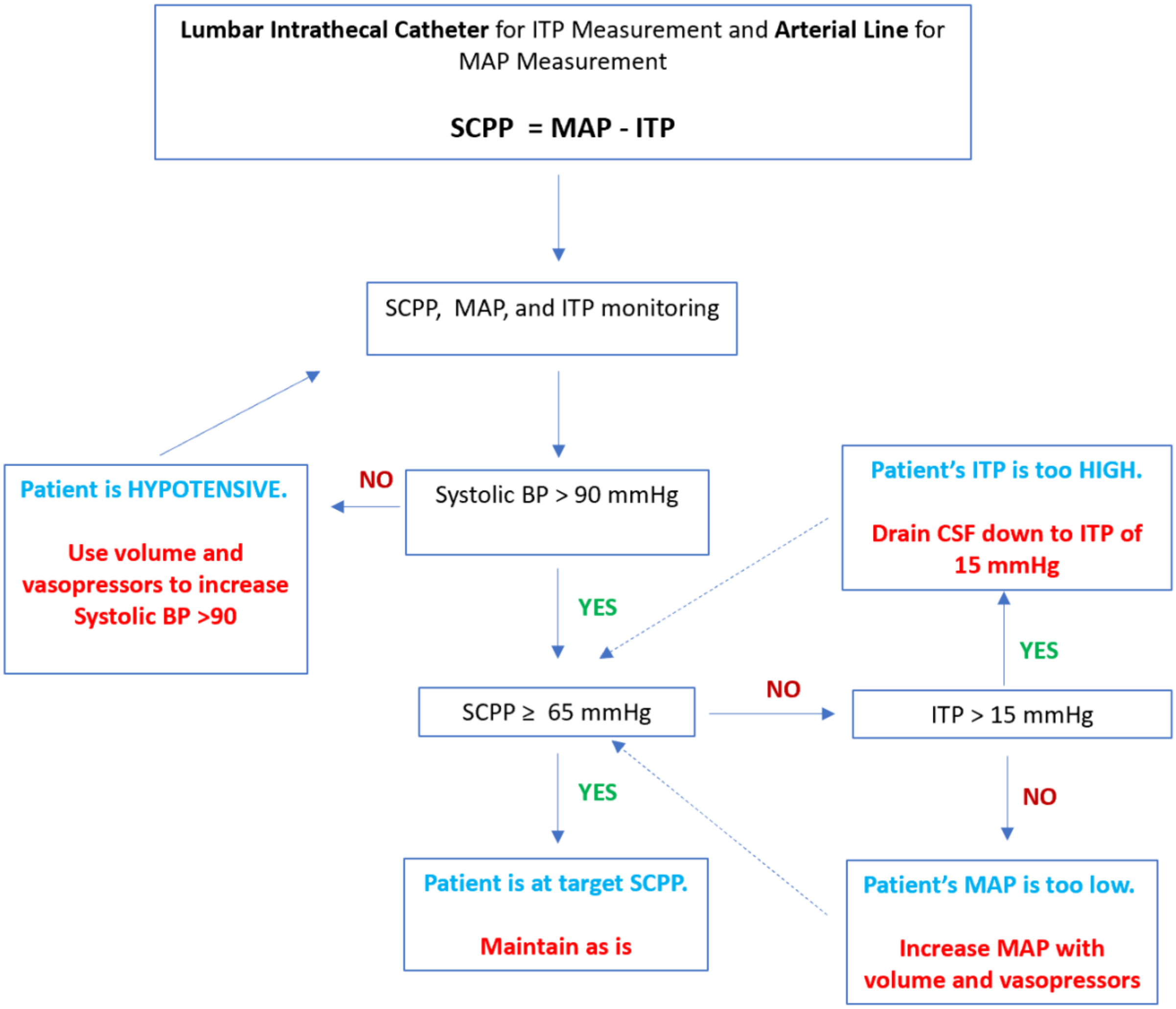

5.4 CSF and Blood Collection and Processing for Biomarker Studies and Biobank Storage

An important aspect of the prospective non-randomized design of the CASPER study is the evaluation of CSF biomarkers to help interpret neurologic recovery and distinguish therapeutic effect from spontaneous recovery. Because participants who are enrolled in this study will have a lumbar intrathecal catheter inserted, there will be the opportunity to obtain CSF samples for biomarker studies. The collection of parallel blood samples for biomarker studies will be achieved through a pre-existing arterial line or central venous catheter.

Sample collection for CSF and blood will occur at the time of lumbar intrathecal catheter insertion and then 3 times per day for the subsequent 7 days that the catheter is in place. A "complete set" of CSF and blood samples will therefore include 21 samples of each biofluid. In an attempt to "standardize" the time of CSF and blood collection, at least one sample per day will be acquired as close as possible to the 24, 48, 72, 96, 120, 144, and 168 hour time point. So, for example, if the subject has the lumbar intrathecal catheter inserted at 19 hrs post-injury, the first CSF sample will be drawn at 19 hrs post-injury, but then another sample will be drawn 5 hours later at 24 hrs post-injury.

In addition, blood samples will be collected at the time of the 3, 6 and 12 month study follow-up visits.

5.4.1. CSF Sampling and Processing

A 6 ml sample of CSF will be obtained at the time of lumbar intrathecal catheter insertion. For subsequent CSF samples obtained post-operatively, a 6 ml sample will be aspirated from the intrathecal catheter.

The 6 ml CSF sample will be divided into 1.5 ml Eppendorf tubes, and then centrifuged at 2000 RCF (or g) for 10 minutes at room temperature. These parameters for CSF centrifugation were recommended in 2014 by the Biomarkers for Alzheimer's and Parkinson's disease (BIOMARKAPD) European multicenter consortium [27]. The supernatant will then be pipetted off in 200 ul increments into pre-labelled 250 ul polypropylene tubes and immediately frozen in an ethanol-dry ice bath and stored at -80°C.

As per the BIOMARKAPD recommendations, it will be endeavored to keep the time between sample acquisition and freezing below 2 hours.

5.4.2. Serum and Plasma Collection and Processing.

At the same time as the CSF sample is collected, a 15 ml sample of blood will be drawn. This will be divided in thirds: 5 ml for serum, 5 ml for plasma, and 5 ml for RNA isolation. The rationale for this division into both serum and plasma is that some analyses may be better suited for serum samples, while others might be better suited for plasma. In order to provide these options for future analyses, the blood sample will be processed for both. Further, a 15 ml blood sample will be collected at the 3, 6 and 12 month study follow-up visit time points.

SERUM

The serum sample will sit at room temperature for 30 minutes. The sample will then be centrifuged at 1200 RCF (g) at room temperature for 20 minutes. The serum will be transferred via pipette into 250 microliter polypropylene vials then frozen at -80°C.

The parameters for serum processing (time of sitting at room temperature and centrifuge settings) are based upon the Standard Operating Procedures for the Early Detection Research Network (EDRN) blood-derived specimen collection protocols.

(https://edrn.nci.nih.gov/resources/standard-operating-procedures/standard-operating-procedures/serum-sop.pdf)

PLASMA

The blood for plasma processing will be added to an EDTA Blood Collection Tube, inverted 10 times, and then centrifuged at 1200 RCF (g) at room temperature for 20 minutes. The plasma layer will be collected by pipette and transferred into 250 microliter polypropylene vials then frozen at -80°C.

The parameters for plasma processing (time of sitting at room temperature and centrifuge settings) are based upon the Standard Operating Procedures for the Early Detection Research Network (EDRN) blood-derived specimen collection protocols.

https://edrn.nci.nih.gov/resources/standard-operating-procedures/standard-operating-procedures/plasma-sop.pdf

RNA ISOLATION

For intracellular RNA isolation, blood will go into a PAXgene blood RNA tube – this will remain at room temperature for a minimum of 2 hours to a maximum of 3 days after collection before transferring to a -20°C freezer. PAXgene samples must stay in -20°C for a minimum of overnight before being moved to final storage at -80°C until shipment. No further processing is required for PAXgene samples at this step.

http://www.bdbiosciences.com/us/applications/blood-collection/cell-biomarker-preservation/paxgenereg-blood-rna-tube/p/762165

The protocol for exactly how to collect blood using the PAXgene blood RNA tube can be found at:

http://static.bdbiosciences.com/documents/PAXgene-RNA-Tube-Blood-Collection-Guide.pdf?_ga=2.140128307.1093169700.1545312660-71292941.1545312660

5.5. Intrathecal Catheter Removal

The intrathecal catheter is to be removed after 7 days or after Sample 21. However, it may be removed prior to 7 days due to technical, clinical or safety concerns at each site (i.e. the clinical team has the discretion to remove the catheter prior to 7 days if it is felt that it should be removed).

The removal process and procedure will be done as per clinical practice standards at each site. It is acknowledged that different sites might have different procedures for how to manage the removal of an intrathecal catheter in a patient who is receiving anticoagulation with low molecular weight heparin. At Vancouver General Hospital, the removal of the catheter will be done according to the 2018 American Society of Regional Anesthesia (ASRA) guidelines, which recommend that *"the catheter should be removed 12 hours after the last dose of LMWH. Subsequent LMWH dosing should occur at least 4 hours after catheter removal"* [28]. In practice, this has been achieved by first identifying when the catheter is to be removed, then identifying a time for the FINAL dose of LMWH at least 12 hours prior to this. This LMWH dose is administered and then an order is written to hold the LMWH until after catheter removal. Within the CAMPER study at VGH, a Pre-Printed Order (PPO) was established to specify the process of monitoring and removal of the intrathecal catheter, which was typically done 20 hours after the last dose of LMWH. Such a PPO will be incorporated into the CASPER study in order to provide clarity to the staff around the management of the catheter.

6. MEASUREMENT OF CLINICAL OUTCOMES

6.1. International Standards for Neurologic Classification of Spinal Cord Injury (ISNCSCI)

For the purposes of ensuring that neurologic deterioration is not occurring while the lumbar intrathecal catheter is in place, an ISNCSCI assessment will be performed daily while the intrathecal catheter is inserted (discussed further in Section 7, Safety). For the purposes of documenting neurologic recovery over time, the ISNCSCI examination will also be performed at 3, 6, and 12 months post-injury. The primary outcomes from this include the single neurological level, AIS Grade and ASIA Motor Score in both the upper and lower extremities.

6.2. Neuropathic Pain – DN4 Questionnaire and International SCI Pain Basic Dataset

For the diagnosis and characterization of neuropathic pain, we will be using the DN4 questionnaire and questionnaires associated with the International SCI Pain Basic Dataset.

The DN4 is a diagnostic tool that is very easy to use but highly discriminating for establishing the presence of neuropathic pain [29]. Typically, a score of 4 or more out of 10 is used as a 'threshold' for diagnosing neuropathic pain.

We have, however, found patients who score "negative" (<4) on the DN4 who we suspect are suffering from neuropathic pain. Hence, we will also administer the questionnaires to assess pain location, type/description, and medications. These questionnaires are aligned with the data elements from the International SCI Pain Basic Dataset, which include average pain intensity and interference over the last 7 days, location of pain, pain medications, brief description of signs and symptoms, and relieving and aggravating factors.

The goal is to perform the DN4 and acquire the SCI Basic Pain Dataset at screening, days 1-7 post-insertion of lumbar intrathecal catheter, 3 months, 6 months and 12 months post-injury for up to 3 different sources of neuropathic pain. The initial administration of the DN4 questionnaire is intended to be performed at the time of enrolment, so that it represents the baseline "neuropathic pain status" of the patient as early as possible. ***Hence, it would be ideal to accrue this data prior to the initiation of any neuropathic pain medication.*** If, for clinical reasons, it is not feasible to administer the questionnaire at the time of patient screening, the investigators should attempt to administer it as soon as possible after the injury. The time at which this first DN4 questionnaire administration occurs should be recorded, and whether the patient is concurrently on neuropathic pain medications at the time should also be documented.

6.3. Spinal Cord Independence Measure (SCIM) III

In order to evaluate overall functional capabilities to conduct activities of daily living (ADL), the Spinal Cord Independence Measure (SCIM) III will be used. The SCIM, now in its third iteration, is a validated, clinician-administered rating scale which assesses 3 areas: self-care, respiration and sphincter management, and mobility (including toileting).

SCIM-III is composed of 20 items in 3 sub-scales:

a. Self-care (6 items, sub-score 0–20);
b. Respiration and sphincter management (4 items, sub-score 0–40); and,
c. Mobility (9 items, sub-score 0–40).

The total score ranges from 0 to 100. Each question describes a specific ADL, and participants who require less assistance, aids, or medical compromise will receive a higher score for those tasks. Items are weighted in terms of their assumed clinical relevance. The SCIM-III is a valid and reliable measure to assess functional recovery in individuals with SCI and will be conducted at 3, 6, and 12 months post-injury.

Schedule of Procedures

Procedure	Enrolment	Catheter Insertion	T 24 h	T 48 h	T 72 h	T 96 h	T 120 h	T 144 h	T 168 h	24 hrs post ITC removal	Month 3[e] (+/- 2 weeks)	Month 6[e] (+/- 2 weeks)	Month 12[e] (+/- 4 weeks)
Informed consent	X												
Eligibility criteria review	X												
Medical history	X												
Demographics	X												
ISNCSCI assessment	X		X	X	X	X	X	X	X		X	X	X
DN4 questionnaire	X[c]		X[c]	X	X	X	X	X	X		X	X	X
Neuropathic pain questionnaires	X[c]		X[c]	X	X	X	X	X	X		X	X	X
CSF samples		X	3X	3X	3X	3X	3X	3X	3X				
Blood samples[a]		X	3X	3X	3X	3X	3X	3X	3X		X	X	X
Biomarker analysis[a]		X	3X	3X	3X	3X	3X	3X	3X				
CSF clinical samples[b]		X	1X	1X	1X	1X	1X	1X	1X				
Shipment of samples to central lab									X[d]				
MAP, ITP, SCPP Monitoring		PERFORMED THROUGHOUT INSERTION OF CATHETER											
SCIM-III											X	X	X
Adverse events		X	X	X	X	X	X	X	X	X (Also 6h after ITC removal)			

a. CSF and blood samples are collected daily at approximately 8-hour intervals from the time of insertion. When possible, one of the scheduled sample collection time points will be adjusted to correspond to the 24, 48, 72, 96, 120, 144, 168 hours post-injury time points (i.e. days 1 through 7 post-injury)

b. CSF cell count, culture (and sensitivity), protein and glucose are clinical assessments required for patients with lumbar drains. These assessments and the timing of them may vary according to hospital-specific protocols. Individual hospital protocols will be followed. At VGH, patients with lumbar drains typically have one "clinical" sample sent each day for analysis in the lab.

c. Baseline pain assessment to be performed prior to administration of medications for neuropathic pain, if possible. Timing will be dependent on ability to assess and ideally will take place as soon as possible following patient enrollment. Date of administration will be recorded as well as whether or not the patient is taking pain medications. Further pain assessments to be performed daily for 7 days following intrathecal catheter (ITC) insertion, again, whenever assessment is physically possible.

d. Shipment of samples to central laboratory (VGH) after removal of lumbar ITC.

e. Study visit windows are included for guidance. Site visits may occur outside these windows. Contact CASPER project manager as needed.

f. Study sites will follow their standard clinical practice in the use of vasopressors.

7. SAFETY / ADVERSE EVENTS

In the previous CAMPER trial, there were no documented cases of CSF leakage, headache, neurologic deterioration, or meningitis attributable to the insertion, presence, or withdrawal of the lumbar intrathecal catheter. **In this CASPER trial, patients will have the intrathecal catheter inserted as part of their clinical management.** However, as part of the clinical follow-up of participants enrolled in CASPER, we will document AEs related to the insertion, presence, or withdrawal of the lumbar intrathecal catheter, and also related to the drainage of CSF to lower ITP. These specific AEs are described further in Section 7.3.

The AE reporting period will be defined as commencing from the time of insertion of the intrathecal catheter until 24 hours after its removal. If an AE is ongoing at the time of catheter removal, it will be monitored until resolution or until 30 days have elapsed.

A review and sign-off by the local Principal Investigator of any AEs related specifically to the presence of the intrathecal catheter will occur for each individual patient at the end of the 24 hours following intrathecal catheter removal. A data safety monitoring board will be established to perform an interim analysis of all complications related to insertion and presence of a lumbar intrathecal catheter and will convene after the first 10 patients enrolled, and then again after 50 patients have been enrolled.

7.1. Defining, Grading and Recording of AEs

7.1.1. Definition of an AE & Serious Adverse Event (SAE)

An **AE** is defined as any untoward medical occurrence in a patient or clinical investigation participant undergoing a medical intervention and which does not necessarily have a causal relationship with the treatment. An AE can therefore be any unfavorable and unintended sign (including an abnormal laboratory finding, for example), symptom, or disease temporally associated with the medical intervention, whether or not it is considered related to the medical intervention. For purposes of this study, AEs will be defined as a change from the patient's baseline that occurs in temporal relationship to any study related procedures (i.e. insertion of the intrathecal catheter or drainage of CSF).

An **SAE** is defined as any untoward medical occurrence that:

- results in death,
- is life-threatening,

  Note: the term "life-threatening" refers to an event in which the patient was at risk of death at the time of the event; it does not refer to an event which hypothetically might have caused death if it were more severe.

- requires inpatient hospitalization or prolongation of existing hospitalization,
- results in persistent or significant disability/incapacity,
- is a congenital anomaly/birth defect,

- is a medically important event or reaction that may not be immediately life-threatening or result in death or hospitalization, but may jeopardize the patient or may require intervention to prevent one of the other outcomes listed above.

Medical and scientific judgment should be exercised in deciding whether expedited reporting is appropriate in other situations, such as important medical events that may not be immediately life-threatening or result in death or hospitalization but may jeopardize the patient, or may require intervention to prevent one of the other outcomes listed in the definition above.

7.1.2. AE Grading

Severity of AEs will be graded according to the National Institute of Health Common Terminology Criteria for Adverse Events (CTCAE), Version 4.0, using the following definitions:

Grades

Grade refers to the severity of the AE. The CTCAE displays Grades 1 through 5 with unique clinical descriptions of severity for each AE based on this general guideline:

- Grade 1 (Mild); asymptomatic or mild symptoms; clinical or diagnostic observations only; intervention not indicated.
- Grade 2 (Moderate); minimal, local or non-invasive intervention indicated; limiting age-appropriate instrumental activities of daily living (ADL).
- Grade 3 (Severe or medically significant but not immediately life-threatening); hospitalization or prolongation of hospitalization indicated; disabling; limiting self-care ADL.
- Grade 4 (Life-threatening consequences); urgent intervention indicated.
- Grade 5; Death related to AE.

7.1.3. Recording of AEs

All AEs that occur during the reporting period, insertion of catheter to 24 hours post-removal, will be recorded in the Adverse Event case report forms. AE data will be obtained from any information volunteered by the patient or through patient questioning. Both the passive system of recording AEs and directed inquiry regarding known potential side effects of insertion and presence of the lumbar intrathecal catheter (see below) will be utilized. Additional information to be recorded includes the **duration** of the AE (onset/resolution dates), **severity**, **relationship** to the insertion and presence of the lumbar intrathecal catheter or drainage of CSF (classified as "probable", "possible", "unlikely" or "unrelated" - see below) and any **concomitant treatment** dispensed (or other action taken). All "serious" and "unexpected" AEs will be reported to the local REB as per local institutional guidelines. Additionally, the 'Serious and Unexpected' Adverse Events will be reported to the CASPER Project Manager.

7.1.4. SAE Reporting

All SAEs that occur during the course of the study must be reported to the CASPER Study Project Manager by telephone within 24 hours of the site becoming aware of the event. When appropriate, the SAE will be documented on the "Serious and Unexpected Adverse Event" case report form and submitted to the CASPER Study Project Manager within 48 hours. All SAEs must be reported to the site's REB according to the local requirements.

7.2. Relationship Between AEs and CSF Drainage or Insertion, Presence, or Withdrawal of the Lumbar Intrathecal Catheter

The relationship between AEs and the insertion and presence of the lumbar intrathecal catheter will be defined as follows:

Probable: A clinical event including, laboratory test abnormality, with a reasonable time sequence to insertion and presence of the lumbar intrathecal catheter unlikely to be attributed to concurrent disease or other drugs or chemicals and which follows a clinically reasonable response on withdrawal (dechallenge). Rechallenge information is not required to fulfill this definition.

Possible: A clinical event, including laboratory test abnormality, with a reasonable time sequence to insertion and presence of the lumbar intrathecal catheter but which could also be explained by concurrent disease or other drugs or chemicals. Information on Intrathecal catheter removal may be unavailable or unclear.

Unlikely: A clinical event, including laboratory test abnormality, with temporal relationship to insertion and presence of the lumbar intrathecal catheter which makes a causal relationship improbable and in which other drugs, chemicals or underlying disease provide plausible explanations.

Unrelated: The clinical event can in no way be related to the insertion or presence of the intrathecal catheter.

7.3. Complications Associated with Lumbar Intrathecal Catheter Insertion or CSF Drainage

As the intrathecal catheter is being inserted as part of the clinical hemodynamic management of the patient, the management of AEs will be left to the discretion of the treating physician. The nature of the AE and the decisions made by the treating physicians will be documented. Below is a description of known complications associated with lumbar catheter insertions and CSF drainage, with estimated incidences.

7.3.1. Post-dural Puncture Headache

The most common risk after lumbar intrathecal catheter insertion is the development of a post-dural puncture headache (PDPH), which may be associated with nausea and vomiting. The incidence of these symptoms has been variably described to be between 2.5% [30] to 59% [31]. ***Because acute SCI patients are not being mobilized into an upright position right away, we expect the risk of this complication to be low in the study population. In the CAMPER study, there were no cases of post-dural puncture headache.***

7.3.2. Neurologic Injury from Lumbar Intrathecal Catheter Insertion or CSF drainage

The placement of the needle (typically a 14-gauge malleable needle) into the lumbar intrathecal space and the insertion of the catheter itself has the potential to injure lumbosacral nerve roots. Grady and colleagues reported on 513 patients in whom CSF drainage was performed while the patient was anesthetized; they found no neurologic deficits [30]. Auroy et al. found 24 cases of neurologic injury in a prospective analysis of 40,640 spinal anesthetics (0.06%) [32]. In a retrospective review of 1.26 million spinal blocks done in Sweden over a decade (1990-1999), Moen et al. identified 56 complications (0.004%) [33]. ***In an extensive review of neurologic complications performed in 2015, ASRA summarized the risk of neuraxial injury to range from 0.001% to 0.07% [34].***

7.3.3. Thromboembolic Prophylaxis for Intrathecal Catheter Insertion and Removal

Patients with complete paralysis are at increased risk of thromboembolic disease. The most recent (2017) clinical practice guidelines from AOSpine recommend the use of low molecular weight heparin (LMWH) to reduce the incidence of thromboembolic disease in SCI patients [35]. The LMWH that will be used for this study is enoxaparin which is administered subcutaneously once daily (40 mg).

Inserting a needle and intrathecal catheter into the epidural space engenders a small risk of epidural hematoma, which is expectedly increased when the patient is anticoagulated. This has therefore incited a number of studies to evaluate the safety of regional spinal anesthesia in the setting of pharmacologic anticoagulation. Practice guidelines have therefore been established based on numerous studies that have documented the safety of regional anesthesia techniques in the patient receiving thromboembolic prophylaxis. The most recent (2018) guidelines set by ASRA and Pain Medicine ASRA specifically address the usage of once daily and twice daily LMWH [28]. The guidelines generally reflect the fact that the increased risk to the patient occurs when the intrathecal catheter is physically either inserted or when it is withdrawn/removed (i.e., when there is a sudden distortion of the epidural space). For this reason, the following has been recommended:

For patients in whom LMWH has begun prior to the insertion of the intrathecal catheter, the guidelines recommend "delay of at least 24 hours prior to needle/catheter placement". Because the lumbar intrathecal catheter is placed within 24 hours of injury, it would be extremely unusual

for a patient to arrive at a specialized trauma centre already on thromboembolic prophylaxis, and thus, we anticipate that this scenario of having to consider patient's coagulation status prior to lumbar intrathecal catheter insertion will occur only very rarely.

The lumbar intrathecal catheter will be placed prior to the surgical procedure for spinal stabilization. The first dose of LMWH will be administered no earlier than 24 hours post-operatively. The ASRA 2018 guidelines recommend that "the catheter should be removed 12 hours after the last dose of LMWH. Subsequent LMWH dosing should occur at least 4 hours after catheter removal" [28].

7.3.4. Meningitis

In Grady's series of 513 patients, one patient reportedly developed meningitis (0.2% incidence) but this was attributed to the transsphenoidal hypophysectomy that the patient had undergone [30]. Cheung and colleagues described two patients out of 162 (1.2%) who developed meningitis after having CSF drainage during thoracoabdominal aortic repairs; one who developed it after the drain was removed, and the other whose catheter fractured off [36]. Coplin and colleagues reviewed 312 patients undergoing lumbar CSF drainage and found an infection rate of 4.2% (13 of 312) [37]. Two-thirds of these patients had already undergone previous CSF drainage procedures or had persistent CSF fistulae, implying that their risk of infection was already higher with a previously "exposed" CSF space. All lumbar drain infections were treated successfully with antibiotic therapy and without neurological deficit. The lumbar intrathecal catheter will remain in situ for up to 7 days for the purpose of intrathecal pressure and spinal cord perfusion pressure monitoring and collection of CSF samples every 8 hours. It is not unreasonable to expect a lower infection rate in SCI patients that have neither CSF fistulae nor a previous drainage procedure. ***The estimated risk of meningitis due to insertion and presence of the lumbar intrathecal catheter is therefore approximately 1%.***

Clinical surveillance of the CSF will be done as per the institutional protocols. At Vancouver General Hospital, a 1 ml sample of CSF is sent daily to the lab for gram stain, cell count, and bacterial culture. We know that the injury itself will induce some meningeal inflammation and elevate the cell count and protein levels; in this regard, the gram stain and bacterial culture will be the critical components of this evaluation. In addition, vital signs of patients will be continuously monitored by nursing staff. A fever of $\geq 38.5^{o}C$ within 24 hours of a positive CSF culture and in the absence of other concurrent infection will be considered to be supportive of the diagnosis of meningitis. If meningitis becomes evident (positive bacterial culture, positive gram stain, and an associated fever of $\geq 38.5^{o}C$), it is expected that the patient will be started on the appropriate antibiotics and the lumbar intrathecal catheter discontinued.

7.3.5. Additional Note on AEs Related to Intrathecal Catheters

The AEs we are particularly concerned with are those that are related to the intrathecal catheters (post-dural puncture headache with associated nausea and vomiting secondary to CSF leakage, neurologic injury due to the insertion of the drain, and meningitis). **In our CAMPER study we did not experience these complications.** In particular, on the issue of meningitis, the risk of this AE would presumably increase the longer that it is left in situ. Catheters were in place for up to 5 days, and no infectious complications related to the catheter occurred. We feel that increasing the time in which the catheter is inserted to 7 days is reasonable, based on our previous 5 day data and on our clinical experience at the Acute Spine Unit at VGH of not having infectious complications with these catheters when they are in place for less than a week. Nonetheless, we will be monitoring the catheters very closely both clinically and with standard CSF measurements to look for signs of meningitis.

8. Biochemical Outcomes & Long Term Storage

8.1. Central Laboratory & Long Term Storage in the International Spinal Cord Injury Biobank (ISCIB)

All CSF and serum samples collected will be sent to, and stored at, a central laboratory facility under the direction of Dr. Brian Kwon at the Blusson Spinal Cord Centre in Vancouver. Full central laboratory contact information and shipping procedures are detailed in the CASPER study manual. Samples will be stored at -80°C in temperature-monitored freezers.

Access to CASPER study freezers is strictly controlled with only authorized CASPER study laboratory personnel having access to study samples. Study samples will be labelled with only the participant's unique study identification number. No personal identifiable information is included on the sample label.

All study samples will be kept until all analyses (as per the study protocol) and publication/presentation of such analysis is completed. After this time, remnant biospecimens (and their corresponding demographic and injury related data) will be stored indefinitely within the **International Spinal Cord Injury Biobank (ISCIB)** and used for future studies under the governance of ISCIB.

8.2. CSF Biomarkers for Injury Stratification and Prediction of Outcome

Based on past work, the CASPER trial will utilize CSF biomarkers to help in the stratification and prediction of outcome. The CSF will be obtained at ~24 hours post-injury from the intrathecal catheter that is installed as part of the hemodynamic management of the patient.

The CSF biomarkers that will be specifically targeted include: IL-6, IL-8, MCP-1, GFAP, S100β, and tau. CSF will be analyzed using a Meso Scale (Meso Scale Discovery, Rockville, MD) multiplex electrochemiluminescent antibody detection assay for IL-6, IL-8, TNF-R1, MCP-1, and IP-10, using a custom 5-plex kit (K15067M, F210V). Plates will be read on the QuickPlex SQ 120 (Meso Scale Discovery), and the data will be analyzed using MSD Discovery Workbench software.

CSF levels of total tau (T-TAU; KHB0042, Invitrogen), S100β (RD192090100R; Biovendor, Modrice, Czech Republic) and GFAP (RD192072200R; Biovendor) will be determined using specific commercially available enzyme-linked immunosorbent assay (ELISA) kits. For each of these individual ELISAs, the assay sensitivities for tau, S100β, and GFAP are 10pg/mL, 15pg/mL, and 45pg/mL, respectively. The intra-assay coefficients of variation (CV) have been less than 5.9, 3.8, and 6.4%, and inter-assay CVs have been less than 9.0, 10.1, and 6.1 for tau, S100β, and GFAP respectively. Concentration levels of these structural proteins were determined based on a standard curve generated on each plate using manufacturer-supplied reagents. For each analyte,

samples have been assayed in duplicate and average concentrations were used for statistical analysis.

Additional biomarker studies for UCH-L1, GFAP, and NF-L will be conducted on CSF and serum using a Quanterix Simoa HD-1 (https://www.quanterix.com/products-technology/ instruments/ hd-1), a fully automated analyzer with the ability to perform a digital ELISA. Simoa improves detection over conventional immunoassays by 1000-3000X by isolating individual capture beads in femtoliter-sized reaction wells [38]. Because array volumes are 2 billion times smaller than conventional assays, only a single molecule is needed to reach the detection limit. This increases the potential of establishing serum biomarkers. This biomarker work will be conducted in collaboration with Dr. Cheryl Wellington, Department of Pathology and Laboratory Medicine, The University of British Columbia. Dr. Wellington is one of the first in Canada to operationalize a Quanterix Simoa in her lab, and is currently collaborating with Dr. Kwon in evaluating these biomarkers on this extremely sensitive platform.

8.3. Biospecimen Storage within ISCIB

CSF and blood samples will be utilized for the biomarker analyses as described above. CSF and blood biospecimens not used in the immediate biomarker analysis will be stored within ISCIB for future analysis. As part of CASPER participation, patients will also be consenting to the long-term storage and secondary analyses of their biospecimens within ISCIB. ISCIB was established by Dr. Brian Kwon who currently is the Director of the Biobank.

ISCIB Mission: The MISSION of the Biobank is to advance the understanding of the biology of SCI through the study of human biospecimens.

ISCIB Vision: The VISION of the Biobank is that it will serve as a resource to the global SCI research community. It is expected that the Biobank would be accessed by investigators from around the world to address research questions about the biology of human SCI that previously may have only been investigated in animal models. It is envisioned that insights into the biology of human SCI, derived from the investigation of biobank specimens, will help to inform the translation of scientific discoveries from “bench to bedside”. Further, such insights will be utilized in a “bedside back to bench” manner to guide subsequent scientific studies in animal models.

ISCIB Objectives: The OBJECTIVE of the Biobank is to support research into the biological responses of human SCI. To do this, the Biobank will share biospecimens with requesting investigators who have established research protocols for biospecimen evaluation.

International Spinal Cord Injury Biobank Governance Structure

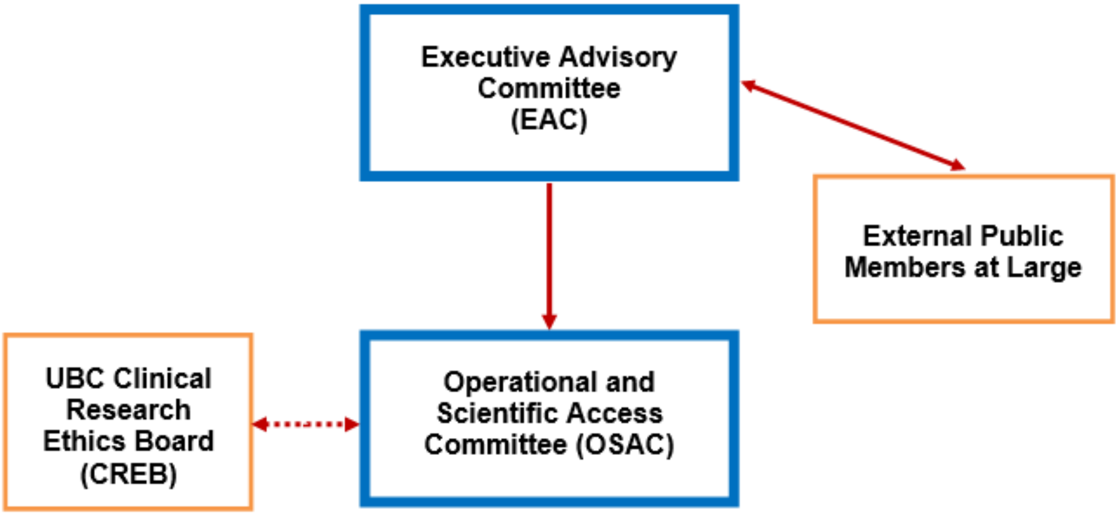

A. ISCIB Executive Advisory Committee (EAC)

The EAC is responsible for the oversight, strategic direction, and overall function of ISCIB. The roles of the EAC include:

- Finalizing and approving the annual report, including the budget.
- Advising strategic direction and priorities.
- Providing guidance around long-term financial sustainability.
- Advocating with the public.
- Maintaining the transparency and ethical nature of biobank operations.
- Assisting the ISCIB Director with decision-making as required.

B. ISCIB Operational and Scientific Access Committee (OSAC)

The OSAC is responsible for the management and operations of the Biobank. Importantly, the OSAC is responsible for decision-making around the usage of biospecimens within the bank. The roles of the OSAC include:

- Establishing and recommending the annual budget.
- Providing oversight and input around the priorities for collection, storage, quantity, quality, and nature of biospecimens.
- Ensuring that the biobank operates according to its declared standards.
- Reviewing requests for biospecimens and deciding upon their distribution in accordance to the mission and vision of ISCIB.
- Reviewing inventory and availability of biospecimens relative to demand.
- Establishing research priorities for access to biospecimens.

9. DATA COLLECTION AND DATA MANAGEMENT

Data collection at each participating study site will be assisted by the use of CASPER study case report forms (CRFs). All data about the participant that is stored at each participating study site, such as CRFs will be coded with a study ID only instead of their name. The documents (electronic or paper) linking the participant's name to the CASPER ID number (e.g., enrollment log, ICF, contact information forms) are only accessible by the local CASPER site research team.

De-identified study data will then be entered into the Global Research Platform (GRP). The GRP is a state-of-the-art, web-based, secure data collection, storage and research management system developed and operated by the Praxis Spinal Cord Institute, formerly the Rick Hansen Institute. The GRP is highly flexible and adaptable, and can be used to run multiple concurrent registries and studies, from local institution clinical data gathering to multicentre clinical trials. GRP is hosted in a top-tier secure data centre in Canada, and meets global privacy and security standards. The GRP is also 21 CFR Part 11 compliant.

User access to data is controlled by study role (i.e. Research Coordinator, Study Manger, Study Monitor, Principal Investigator), the study and the facility to which the user belongs. To access the GRP, the user requires a valid username and password, and a certificate installed on the user's computer that expires and needs to be renewed annually. The certificate is issued when a valid user account has been created.

As a multicenter study, participating CASPER study sites will only see data entered from their own study site. As the principal investigator, Dr. Brian Kwon at the University of British Columbia and his delegated research team members will have access to the de-identified study data provided by all of the participating study sites.

CASPER data will be analyzed as described in the protocol and only de-identified data will be included in reports and publications.

10. EFFICACY ENDPOINT

The primary purpose of the CASPER trial is to determine if active management of SCPP results in improved neurologic recovery at 6 months post-injury as compared to conventional hemodynamic management.

Neurologic recovery will be measured using the ISNCSCI examination. Our primary outcome measure will be the change in total motor score at 6 months post-injury (a time point at which most motor recovery has occurred and is commonly used in acute SCI clinical trials). To assess whether active SCPP management (SCPP ≥ 65 mmHg x 7 days) promotes better neurologic recovery than conventional MAP management, our **control group will be the prospectively collected (and conventionally managed) cohort from our CAMPER study**. The major

advantage of this CAMPER cohort is that it represents a well-documented & prospectively collected set of neurologic, hemodynamic, and biomarker data.

Sample Size Calculation.

The most conventional approaches to establishing sample size consider either AIS grade conversion or motor score improvement. Here, we have considered both AIS grade conversion and motor score improvement. If we were to consider “AIS Grade Conversion” to be our primary outcome measure, and address the question of whether active SCPP management promoted AIS grade improvement as compared to conventional MAP management, then using the clinical grading alone, a 10% improvement would require an n=143 for 80% power, based upon our previous publication in which we evaluated AIS grade improvement as predicted by either the baseline AIS grade, MRI, or CSF biomarkers [20]. However, if using the CSF biomarkers to predict outcome, a 10% improvement would require an n=72 for 80% power. Because we recognize that the CSF biomarkers will help in predicting outcome, but that the strength of this prediction will likely be less in a completely independent cohort of patients, we do not feel that reducing the sample size nearly in half (from 143 to 72) is prudent. Hence, we propose an **n=100**, assuming a more conservative improvement in our ability to predict outcome with the CSF biomarkers.

Secondarily, if we view a 7-point improvement in motor score as both clinically meaningful/achievable, then we require the enrolment of ~74 cervical SCI patients into this proposed study, based upon a standard deviation of 11 points, reflecting α=0.0499 and β=0.806. A 7-point motor score change is chosen because this “effect size” is what appears to be achievable with early surgical decompression in acute SCI [39, 40]. Assuming that we will also include thoracic SCI where motor score improvement is not as responsive due to the inability to assess segmental motor recovery, we feel that enrolling a total of 100 patients would be more sufficient in this study.

Analysis Plan. We will use multivariable linear and logistic regression to model the association between the intervention (SCPP management vs. MAP management) and neurologic outcome (motor score improvement or rate of AIS grade improvement). Anticipated co-variates to control for include AIS grade, neurologic level, baseline motor score, 24 hr post-injury CSF biomarker levels, age, and gender. As a sensitivity analysis we will utilize propensity score matching, a statistical approach that we have employed previously in an assessment of methylprednisolone for acute SCI [41].

Study Design Considerations.

One question worth addressing is ***“why not just do a PRCT of active SCPP management versus MAP augmentation?”*** There are two practical issues to consider. Firstly, conducting a PRCT in acute SCI is a massive undertaking. Only four pivotal acute SCI PRCTs have ever been successfully completed; the last one (Sygen) finishing almost 20 years ago [42-45]. Our current experience indicates that it has not gotten much easier. Consider that enrolment in the Phase 3 PRCT for Riluzole in acute cervical SCI led by Dr. Fehlings began in 2013, and despite having over

participating 20 sites, enrolment will likely not finish until 2021. The Phase 2 PRCT of minocycline for SCI took over 4 years to recruit 51 patients [46]. Additional Phase 2 acute SCI trials of a MgCl formulation and a βFGF analogue were terminated due to poor enrolment. So, while a PRCT design has obvious advantages, these practical realities must be carefully considered before embarking upon a PRCT in acute SCI.

Secondly, if we were to conduct a PRCT, the "treatment arm" would have intrathecal catheters inserted and active SCPP management to keep SCPP ≥ 65 mmHg, while the "control arm" would have intrathecal catheters inserted and routine MAP augmentation. **However, this "control arm" is exactly what our CAMPER study cohort consists of** – patients who had an intrathecal catheter placed but then routine hemodynamic management based on MAP. Given that we possess this prospectively collected CAMPER cohort with well-documented neurologic and hemodynamic data, there is a practical rationale to incorporate this cohort into our proposed study, utilizing contemporary statistical methods to control for confounding variables. One of the confounding variables that we will uniquely be able to control for is the levels of the CSF biomarkers that we have also already collected in our CAMPER patients.

REFERENCES

1. Medicine, C.f.S.C., *Early acute management in adults with spinal cord injury: a clinical practice guideline for health-care professionals*. J Spinal Cord Med. Vol. 31. 2008. 77.
2. Ploumis, A., et al., *A systematic review of the evidence supporting a role for vasopressor support in acute SCI.* Spinal Cord, 2010. **48**(5): p. 356-62.
3. Casha, S. and S. Christie, *A systematic review of intensive cardiopulmonary management after spinal cord injury.* J Neurotrauma, 2011. **28**(8): p. 1479-95.
4. Ryken, T.C., et al., *The acute cardiopulmonary management of patients with cervical spinal cord injuries.* Neurosurgery, 2013. **72 Suppl 2**: p. 84-92.
5. Hawryluk, G., et al., *Mean Arterial Blood Pressure Correlates with Neurological Recovery after Human Spinal Cord Injury: Analysis of High Frequency Physiologic Data.* J Neurotrauma, 2015.
6. Kong, C.Y., et al., *A prospective evaluation of hemodynamic management in acute spinal cord injury patients.* Spinal Cord, 2013. **51**(6): p. 466-71.
7. Khan, N.R., et al., *The use of lumbar drains in preventing spinal cord injury following thoracoabdominal aortic aneurysm repair: an updated systematic review and meta-analysis.* Journal of Neurosurgery: Spine, 2016. **25**(3): p. 383-393.
8. Awad, H., et al., *Spinal cord injury after thoracic endovascular aortic aneurysm repair.* Can J Anaesth, 2017. **64**(12): p. 1218-1235.
9. Etz, C.D., et al., *Contemporary spinal cord protection during thoracic and thoracoabdominal aortic surgery and endovascular aortic repair: a position paper of the vascular domain of the European Association for Cardio-Thoracic Surgerydagger.* Eur J Cardiothorac Surg, 2015. **47**(6): p. 943-57.
10. Kwon, B.K., et al., *Intrathecal pressure monitoring and cerebrospinal fluid drainage in acute spinal cord injury: a prospective randomized trial.* J Neurosurg Spine, 2009. **10**(3): p. 181-93.
11. Squair, J.W., et al., *Spinal cord perfusion pressure predicts neurologic recovery in acute spinal cord injury.* Neurology, 2017. **89**(16): p. 1660-1667.
12. Lee, R.S., et al., *Feasibility of patient recruitment into clinical trials of experimental treatments for acute spinal cord injury.* J Clin Neurosci, 2012. **19**(10): p. 1338-43.
13. Fehlings, M.G., et al., *Early versus delayed decompression for traumatic cervical spinal cord injury: results of the Surgical Timing in Acute Spinal Cord Injury Study (STASCIS).* PLoS One, 2012. **7**(2): p. e32037.
14. Fawcett, J.W., et al., *Guidelines for the conduct of clinical trials for spinal cord injury as developed by the ICCP panel: spontaneous recovery after spinal cord injury and statistical power needed for therapeutic clinical trials.* Spinal Cord, 2007. **45**(3): p. 190-205.
15. Streijger, F., et al., *A Targeted Proteomics Analysis of Cerebrospinal Fluid after Acute Human Spinal Cord Injury.* J Neurotrauma, 2017.
16. Kwon, B.K., et al., *Cerebrospinal Fluid Biomarkers To Stratify Injury Severity and Predict Outcome in Human Traumatic Spinal Cord Injury.* J Neurotrauma, 2017. **34**(3): p. 567-580.

17. Wu, Y., et al., *Parallel Metabolomic Profiling of Cerebrospinal Fluid and Serum for Identifying Biomarkers of Injury Severity after Acute Human Spinal Cord Injury.* Sci Rep, 2016. **6**: p. 38718.
18. Pouw, M.H., et al., *Structural biomarkers in the cerebrospinal fluid within 24 h after a traumatic spinal cord injury: a descriptive analysis of 16 subjects.* Spinal Cord, 2014. **52**(6): p. 428-33.
19. Kwon, B.K., et al., *Cerebrospinal fluid inflammatory cytokines and biomarkers of injury severity in acute human spinal cord injury.* J Neurotrauma, 2010. **27**(4): p. 669-82.
20. Dalkilic, T., et al., *Predicting Injury Severity and Neurological Recovery after Acute Cervical Spinal Cord Injury: A Comparison of Cerebrospinal Fluid and Magnetic Resonance Imaging Biomarkers.* J Neurotrauma, 2018. **35**(3): p. 435-445.
21. Readdy, W.J., et al., *Complications and outcomes of vasopressor usage in acute traumatic central cord syndrome.* J Neurosurg Spine, 2015: p. 1-7.
22. Inoue, T., et al., *Medical and surgical management after spinal cord injury: vasopressor usage, early surgerys, and complications.* J Neurotrauma, 2014. **31**(3): p. 284-91.
23. Saadoun, S. and M.C. Papadopoulos, *Spinal cord injury: is monitoring from the injury site the future?* Crit Care, 2016. **20**(1): p. 308.
24. Aarabi, B., et al., *Extent of Spinal Cord Decompression in Motor Complete (American Spinal Injury Association Impairment Scale Grades A and B) Traumatic Spinal Cord Injury Patients: Post-Operative Magnetic Resonance Imaging Analysis of Standard Operative Approaches.* J Neurotrauma, 2018.
25. Altaf, F., et al., *The differential effects of norepinephrine and dopamine on cerebrospinal fluid pressure and spinal cord perfusion pressure after acute human spinal cord injury.* Spinal Cord, 2016.
26. Streijger, F., et al., *A Direct Comparison Between Norepinephrine and Phenylephrine for Augmenting Spinal Cord Perfusion in a Porcine Model of Spinal Cord Injury.* J Neurotrauma, 2018.
27. Teunissen, C.E., *Biobanking of CSF: international standardization to optimize biomarker development.* Clinical biochemistry. **47**(4-5): p. 288-292.
28. Horlocker, T.T., et al., *Regional Anesthesia in the Patient Receiving Antithrombotic or Thrombolytic Therapy: American Society of Regional Anesthesia and Pain Medicine Evidence-Based Guidelines (Fourth Edition).* Reg Anesth Pain Med, 2018. **43**(3): p. 263-309.
29. Calmels, P., et al., *Neuropathic pain in spinal cord injury: identification, classification, evaluation.* Ann Phys Rehabil Med, 2009. **52**(2): p. 83-102.
30. Grady, R.E., et al., *Neurologic complications after placement of cerebrospinal fluid drainage catheters and needles in anesthetized patients: implications for regional anesthesia. Mayo Perioperative Outcomes Group.* Anesth Analg, 1999. **88**(2): p. 388-92.
31. Roland, P.S., et al., *Complications of lumbar spinal fluid drainage.* Otolaryngol Head Neck Surg, 1992. **107**(4): p. 564-9.
32. Auroy, Y., et al., *Serious complications related to regional anesthesia: results of a prospective survey in France.* Anesthesiology, 1997. **87**(3): p. 479-86.
33. Moen, V., N. Dahlgren, and L. Irestedt, *Severe neurological complications after central neuraxial blockades in Sweden 1990-1999.* Anesthesiology, 2004. **101**(4): p. 950-9.

34. Neal, J.M., et al., *The Second ASRA Practice Advisory on Neurologic Complications Associated With Regional Anesthesia and Pain Medicine: Executive Summary 2015.* Reg Anesth Pain Med, 2015. **40**(5): p. 401-30.
35. Fehlings, M.G., et al., *A Clinical Practice Guideline for the Management of Patients With Acute Spinal Cord Injury: Recommendations on the Type and Timing of Anticoagulant Thromboprophylaxis.* Global Spine J, 2017. **7**(3 Suppl): p. 212s-220s.
36. Cheung, A.T., et al., *Safety of lumbar drains in thoracic aortic operations performed with extracorporeal circulation.* Ann Thorac Surg, 2003. **76**(4): p. 1190-6; discussion 1196-7.
37. Coplin, W.M., et al., *Bacterial meningitis associated with lumbar drains: a retrospective cohort study.* J Neurol Neurosurg Psychiatry, 1999. **67**(4): p. 468-73.
38. Haber, M., et al., *Defining an Analytic Framework to Evaluate Quantitative MRI Markers of Traumatic Axonal Injury: Preliminary Results in a Mouse Closed Head Injury Model.* eNeuro, 2017. **4**(5).
39. Lenehan, B., et al., *The urgency of surgical decompression in acute central cord injuries with spondylosis and without instability.* Spine (Phila Pa 1976), 2010. **35**(21 Suppl): p. S180-6.
40. van Middendorp, J.J., A.J. Hosman, and S.A. Doi, *The effects of the timing of spinal surgery after traumatic spinal cord injury: a systematic review and meta-analysis.* J Neurotrauma, 2013. **30**(21): p. 1781-94.
41. Evaniew, N., et al., *Methylprednisolone for the Treatment of Patients with Acute Spinal Cord Injuries: A Propensity Score-Matched Cohort Study from a Canadian Multi-Center Spinal Cord Injury Registry.* J Neurotrauma, 2015.
42. Bracken, M.B., et al., *Efficacy of methylprednisolone in acute spinal cord injury.* Jama, 1984. **251**(1): p. 45-52.
43. Bracken, M.B., et al., *A randomized, controlled trial of methylprednisolone or naloxone in the treatment of acute spinal-cord injury. Results of the Second National Acute Spinal Cord Injury Study.* N Engl J Med, 1990. **322**(20): p. 1405-11.
44. Bracken, M.B., et al., *Administration of methylprednisolone for 24 or 48 hours or tirilazad mesylate for 48 hours in the treatment of acute spinal cord injury. Results of the Third National Acute Spinal Cord Injury Randomized Controlled Trial. National Acute Spinal Cord Injury Study.* Jama, 1997. **277**(20): p. 1597-604.
45. Geisler, F.H., et al., *The Sygen multicenter acute spinal cord injury study.* Spine (Phila Pa 1976), 2001. **26**(24 Suppl): p. S87-98.
46. Casha, S., et al., *Results of a phase II placebo-controlled randomized trial of minocycline in acute spinal cord injury.* Brain, 2012. **135**(Pt 4): p. 1224-36.
