# Supplementary material for: Targeting spinal cord perfusion pressure in acute spinal cord injury through cerebrospinal fluid drainage: A prospective multi-center clinical trial
Source: PLoS Med. 2026 Feb 5;23(2):e1004925. doi: 10.1371/journal.pmed.1004925 (PMC12890222; doi:10.1371/journal.pmed.1004925)
Supplement: S2 Consort Checklist — (DOCX) [file pmed.1004925.s007.docx]

| Item | Description | Reported on line no |
| --- | --- | --- |
| Title | Identification of the study as randomized | 3-4 (Not Randomized) |
| Authors | Contact details for corresponding author | 8-36 |
| Trial Design | Description of the trial design (e.g. parallel, cluster, non-inferiority) | 4,59 |
| Methods |  |  |
| Participants | Eligibility criteria for participants and the settings where the data were collected | 57 |
| Interventions | Interventions intended for each group | 59-63 |
| Objectives | Specific objective or hypothesis | 55-56 |
| Randomization | How participants were allocated to interventions | N/A |
| Blinding (masking) | Whether or not participants, care givers, and those assessing the outcomes were blinded to group assignment | N/A |
| Results |  |  |
| Numbers randomized | Number of participants randomized to each group | Not Randomized |
| Recruitment | Trial status | 61, 63 |
| Numbers analyzed | Number of participants analyzed in each group | 57, 60-61 |
| Outcome | For the primary outcome, a result for each group and the estimated effect size and its precision | 72-73 |
| Harms | Important adverse events or side effects | 76-77 |
| Conclusions | General interpretation of the results | 78-84 |
| Trial Registration | Registration number and name of trial register | 60 |
| Funding | Source of funding | Reported in submission portal. |

**CONSORT Checklist for Abstracts**

Note1: Table re-created based on information provided in Table 1 at <https://journals.plos.org/plosmedicine/article?id=10.1371/journal.pmed.0050020>

Note2: The trial was not randomized, therefore some items are not addressed.
